# Soluble Urokinase Plasminogen Activator Receptor Predicts Survival and Hepatic Decompensation in Advanced Hepatocellular Carcinoma

**DOI:** 10.1111/liv.70121

**Published:** 2025-05-02

**Authors:** Fabian Artusa, Sven Lamatsch, Minh Duc Phan, Burcin Özdirik, Hilmar Berger, Mara Egerer, Jana Knorr‐Klocke, Janett Fischer, Rhea Veelken, Florian van Bömmel, Thomas Berg, Kai Kappert, Rudolf Tauber, Tobias Puengel, Cornelius Engelmann, Münevver Demir, Frank Tacke, Raphael Mohr

**Affiliations:** ^1^ Department of Hepatology and Gastroenterology Charité ‐ Universitätsmedizin Berlin, Campus Virchow Klinikum (CVK) and Campus Charité Mitte (CCM) Berlin Germany; ^2^ Division of Hepatology, Department of Medicine II Leipzig University Medical Center Leipzig Germany; ^3^ Institute of Diagnostic Laboratory Medicine, Clinical Chemistry and Pathobiochemistry Charité ‐ Universitätsmedizin Berlin Berlin Germany; ^4^ Labor Berlin – Charité Vivantes GmbH Berlin Germany; ^5^ Institute for Liver and Digestive Health University College London London UK

**Keywords:** atezolizumab, bevacizumab, biomarker, hepatocellular carcinoma (HCC), soluble urokinase plasminogen activator receptor (suPAR), survival

## Abstract

**Background and Aims:**

The introduction of immune checkpoint inhibitor (ICI) based therapies has significantly improved the prognosis of patients with unresectable hepatocellular carcinoma (HCC). However, the variable treatment response and the uncertain benefit in patients with advanced liver cirrhosis emphasise the urgent need for prognostic and predictive biomarkers guiding patient selection. The soluble urokinase plasminogen activator receptor (suPAR) is strongly associated with inflammation, liver cirrhosis and various types of cancer. In this study, we investigated suPAR as a potential novel biomarker in patients with unresectable HCC.

**Methods:**

This multicenter retrospective study, conducted at three German tertiary care centers, included 90 patients with unresectable HCC and suPAR measurements prior to and during atezolizumab/bevacizumab therapy. Patients with liver cirrhosis without HCC (*n* = 235) and non‐cirrhotic patients with other gastrointestinal tumours (*n* = 155) were selected as control cohorts.

**Results:**

Median suPAR levels were significantly higher in patients with liver cirrhosis compared to non‐cirrhotic cancer patients. A strong association with parameters of liver function, but not with HCC characteristics, was observed. In patients with HCC receiving atezolizumab/bevacizumab, suPAR was the most accurate independent predictor of hepatic decompensation and overall survival (OS). In addition, suPAR was able to stratify the risk of hepatic decompensation within the different Child‐Pugh classes.

**Conclusions:**

SuPAR represents a promising novel biomarker in patients with HCC treated with ICI‐based therapies and bears the potential to guide the selection of antitumoral systemic therapies in patients with advanced liver cirrhosis.

**Abbreviations**
ACLF: acute on chronic liver failure
AFP: alpha‐fetoprotein
ALBI score: albumin–bilirubin score
ALD: alcohol‐associated liver disease
AUC: area under the curve
BCLC: Barcelona Clinic Liver Cancer
CRP: C‐reactive protein
ECOG: Eastern Cooperative Oncology Group
ESMO: European Society of Medical Oncology
GEP‐NEN: gastroenteropancreatic neuroendocrine neoplasia
HCC: hepatocellular carcinoma
HR: hazard ratio
ICI: immune checkpoint inhibitor
INR: international normalised ratio
IQR: interquartile range
MELD: model for end‐stage liver disease
NEC: neuroendocrine carcinoma
NET: neuroendocrine tumours
ORR: objective response rate
OS: overall survival
PFS: progression‐free survival
RECIST: Response Evaluation Criteria in Solid Tumours
ROC: receiver operating characteristic
(s)uPAR: (soluble) urokinase plasminogen activator receptor

**Summary**

- Immunotherapy is the standard of care for patients with advanced liver cancer. However, not all patients benefit from immunotherapy.
- SuPAR is strongly associated with inflammation, liver function and various types of cancer.
- Here we report that suPAR independently predicts hepatic decompensation and overall survival in liver cancer patients and may serve as a novel biomarker guiding treatment decisions.

## 1 Introduction

Hepatocellular carcinoma (HCC), the most common form of primary liver cancer, poses a global health burden with its increasing incidence and limited therapeutic options [1, 2]. The combination of atezolizumab, an ICI targeting programmed death‐ligand 1, and bevacizumab, a vascular endothelial growth factor inhibitor, has demonstrated remarkable efficacy in clinical trials and represents one standard of care option for advanced HCC [3, 4, 5, 6, 7]. However, the overall prognosis of advanced HCC remains poor.

Impaired liver function represents a major prognostic factor for overall survival (OS) in patients with HCC, and deteriorates the efficacy and tolerability of systemic treatments [8, 9, 10, 11, 12]. Although comparable objective response rates (ORR) and manageable safety profiles have been described for Child‐Pugh B patients, OS is significantly inferior compared to Child‐Pugh A patients [8, 10, 13]. Competing effects of cirrhosis‐related complications versus tumour progression may contribute to this observation [14]. There is an urgent need for biomarkers that predict treatment response and likelihood of hepatic decompensation to better guide patient selection and identify patients with advanced liver cirrhosis who benefit from immunotherapy.

The soluble urokinase plasminogen activator receptor (suPAR) is the circulating form of the membrane‐bound urokinase plasminogen activator receptor (uPAR, CD87). uPAR is expressed by a variety of cells, including immune cells (e.g., monocytes, macrophages and neutrophils), endothelial cells and fibroblasts [15, 16, 17]. It represents a key mediator of plasminogen activation and extracellular matrix proteolysis and is involved in proliferation, migration, adhesion and angiogenesis [16, 18]. The release of suPAR is stimulated during inflammation, injury, or stress [17, 18]. Due to its temporal and kinetic stability with only minimal affection by short‐term influences, suPAR has been suggested as a biomarker for chronic inflammation [17]. uPAR is also expressed in a broad range of cancers and might serve as a biomarker in the context of different inflammatory and malignant diseases [19, 20, 21, 22, 23, 24, 25, 26].

Furthermore, suPAR levels have been demonstrated to correlate with liver inflammation, fibrosis and cirrhosis in patients with chronic liver disease [27, 28, 29]. In patients with decompensated cirrhosis, suPAR levels were associated with organ failure and poor short‐term survival [30, 31, 32]. Since persistent immune activation is a central driver of hepatic decompensation and further organ dysfunction [33, 34, 35], the approach of mapping the hepatic and systemic inflammation level before ICI‐based therapy appears promising. Nevertheless, data on suPAR as a potential biomarker in the context of unresectable HCC treated with ICI are lacking.

Therefore, in the present study, we analysed the performance of suPAR as a predictive and prognostic biomarker for OS, hepatic decompensation and treatment response in a well‐characterised cohort of patients with unresectable HCC receiving immunotherapy with atezolizumab/bevacizumab.

## 2 Methods

### 2.1 Patients and Data Collection

This retrospective multicentre study included 90 patients with advanced HCC and available suPAR measurement within 1 month prior to atezolizumab/bevacizumab initiation at two tertiary centres for gastrointestinal oncology at the Charité—Universitätsmedizin Berlin between September 2020 and March 2023. Patients with liver function Child‐Pugh A and B, as well as performance status between 0 and 2 on the Eastern Cooperative Oncology Group (ECOG) performance status scale were considered eligible for systemic therapy with atezolizumab/bevacizumab. The diagnosis of HCC was confirmed according to the European Society of Medical Oncology (ESMO) criteria by histologic analysis or clinical and imaging features for patients with liver cirrhosis [36].

To distinguish the influence of different stages of liver cirrhosis and HCC on circulating suPAR levels, two control cohorts were formed. The first control group contained 235 patients with liver cirrhosis Child‐Pugh A‐C without evidence of malignant disease or acute on chronic liver failure (ACLF) who were treated at the Leipzig University Hospital (*n* = 127) or the Charité—Universitätsmedizin Berlin (*n* = 108) between January 2020 and March 2024. The presence of cirrhosis was determined by histopathological workup or non‐invasive assessment of liver stiffness and typical findings on imaging, laboratory values and medical history. Advanced liver cirrhosis was defined as a Child‐Pugh score of B or C.

As a second non‐cirrhotic cancer control cohort, 157 therapy‐naïve patients with gastroenteropancreatic neuroendocrine neoplasia (GEP‐NEN), who were treated at the Charité—Universitätsmedizin Berlin between 2012 and 2020, were selected [15]. The presence of GEP‐NEN was confirmed by histopathological workup. Patients were categorised according to the histological grading and the Ki‐67 proliferative index into neuroendocrine tumours (NET) and neuroendocrine carcinomas (NEC).

The present study complies with the Declaration of Helsinki and was approved by the local ethics committee (EA2/091/19).

### 2.2 Treatment Regimes in HCC Patients

Patients with HCC received as a first‐line systemic treatment a fixed dose of 1200 mg atezolizumab plus 15 mg bevacizumab per kilogram body weight intravenously Q3W according to the IMbrave 150 trial [37]. Treatment was continued until disease progression or unacceptable toxicity. Tumours were assessed by magnetic resonance imaging and/or computed tomography at baseline and every 9–12 weeks thereafter. Imaging response assessment was performed according to the current response evaluation criteria in solid tumours (RECIST v1.1) [38, 39].

### 2.3 Clinical and Laboratory Assessment

Data on baseline characteristics and outcomes were taken from the respective hospital's electronic patient records. The collected data included baseline demographics, as well as clinical and laboratory parameters of liver function. For patients with HCC, ECOG performance status, tumour characteristics, alpha‐fetoprotein (AFP) values, treatment response and severe adverse events were recorded. Body weight, abdominal girth increase, changes in clinical symptoms, as well as hospitalisations were recorded systematically using a questionnaire at every visit or before each therapy application.

### 2.4 Measurement of Circulating suPAR Levels

Levels of suPAR were measured using suPARnostic TurbiLatex (ViroGates, Birkerød, Denmark) according to the manufacturers' instructions. The measurement of all samples was performed by the central laboratory of Charité—Universitätsmedizin Berlin, Labor Berlin—Charité Vivantes, in a semi‐automated fashion, using Cobas 8000 Module 701c (limit of detection 1.3 ng/mL, linearity from 1.8 ng/mL to 26.5 ng/mL). Plasma was retrieved using centrifugation at 2000 rpm for 10 min at room temperature. To avoid repeated freeze‐thawing, aliquots were snap‐frozen at −80°C until further use.

### 2.5 Study Objectives and Endpoints

The objective was to investigate the prognostic performance of suPAR in patients with HCC receiving atezolizumab/bevacizumab with regard to OS, ORR and time without hepatic decompensation. Hepatic decompensation was defined as new or progressive ascites requiring hospitalisation, new or worsening hepatic encephalopathy requiring hospitalisation and/or variceal bleeding. Additionally, suPAR was tested as a predictor of laboratory deterioration of liver function. Therefore, absolute and relative laboratory value changes were analysed. Furthermore, we hypothesised that the prognostic performance of suPAR might differ between cirrhotic patients with versus without HCC, and might differ between cancer patients with vs. without liver cirrhosis. OS was calculated from the day of suPAR blood collection until death. Patients were followed up until July 2023. When patients were lost to follow‐up, they were censored at the time of the last contact. Patients who underwent a liver transplantation were censored on the day of transplantation.

### 2.6 Statistical Analysis

Data are expressed as median with interquartile range (IQR) or frequencies and percentages. Discrete variables were compared using Fisher's exact test, and continuous variables with the Mann–Whitney *U* test for independent samples. Kruskal–Wallis ANOVA and Mann–Whitney *U* test were applied for post hoc analysis for the comparison of more than two groups. Kaplan–Meier analysis was applied to OS, progression‐free survival (PFS), time to adverse events grade 3 or 4 and time to hepatic decompensation. Differences between groups were assessed with the log‐rank test. Hazard ratios (HR) for death, adverse events and hepatic decompensation were estimated with a stratified Cox proportional‐hazards model (backward stepwise likelihood‐quotient) and confirmed by bootstrapping. Baseline variables with *p* < 0.10 at the univariate analysis were included in the multivariate model. The area under the receiver operating characteristic (ROC) curve (AUC) was calculated to evaluate the prognostic performance of laboratory parameters at baseline and at monthly sampling, as well as of their absolute and relative changes to predict hepatic decompensation and mortality. All probability values were 2‐tailed and considered significant when *p* < 0.05 (*p < 0.05; **p < 0.01; ***p < 0.001). Data analysis was performed using IBM SPSS statistics 27 and visualised using GraphPad Prism 10.2 (GraphPad Software, San Diego, CA, USA). Time‐dependent AUC was performed using the R‐package timeROC with bootstrapped confidence intervals [40].

## 3 Results

### 3.1 Patient Characteristics

Within the HCC cohort, 64% of patients were Barcelona Clinic Liver Cancer (BCLC) stage C, 70% had non‐viral HCC, 21% had advanced liver cirrhosis Child‐Pugh B and 32% had a reduced ECOG performance status ≥ 1. Full baseline characteristics of all HCC patients are shown in Table S1. When compared with the cirrhotic control group, HCC patients were older and showed a higher frequency of male gender, viral aetiology and compensated liver cirrhosis Child‐Pugh A (Table 1). When compared with the non‐cirrhotic cancer control cohort, HCC patients were older (median age 67 [95% CI, 60–72] vs. 60 [95% CI, 48–68], *p* < 0.001), showed a higher frequency of male sex (86% vs. 49%, *p* < 0.001) and had less frequent distant metastasis (34% vs. 82%, *p* < 0.001). Baseline characteristics of patients with NET versus NEC are shown in Table S2. Liver metastases were present in 58% and 60%, respectively.

**TABLE 1:** Baseline patient characteristics.

	Cirrhosis with HCC	Cirrhosis without HCC	*p*
	(*n* = 90)	(*n* = 235)	
Median age, median (IQR)—year	67 (60–72)	58 (50–65)	< 0.001
Male sex, *n* (%)	77 (86)	140 (60)	< 0.001
BMI median (IQR)—kg/m^2^	27 (24–30)	27 (24–32)	0.523
Child–Pugh classification, *n* (%)			
A5‐6	71 (79)	122 (52)	< 0.001
B7‐9	19 (21)	85 (37)	< 0.001
C10	0 (0)	28 (12)	< 0.001
Relevant comorbidities, no. (%)			
Chronic kidney disease	20 (22)	12 (24)	0.836
Laboratory parameters			
MELD score, median (IQR)	8 (7–10)	11 (8–15)	< 0.001
Bilirubin, median (IQR)—mg/dL	0.6 (0.5–1.3)	1.0 (0.6–2.4)	< 0.001
Creatinine, median (IQR)—mg/dL	0.9 (0.8–1.1)	0.8 (0.7–1.0)	0.179
INR, median (IQR)	1.1 (1.0–1.2)	1.2 (1.1–1.3)	< 0.001
Albumin, median (IQR)—g/L	4.0 (3.5–4.3)	3.7 (3.4–4.1)	0.045
ALBI score, median (IQR)	−2.6 (−2.9 to −2.2)	−2.3 (−2.8 to −1.7)	0.005
SuPAR, median (IQR)	10.5 (7.1–13.3)	11 (7.1–14.9)	0.131
Aetiology of liver cirrhosis, *n* (%)			
Hepatitis B	17 (19)	11 (5)	< 0.001
Hepatitis C	10 (11)	16 (7)	0.238
Alcohol‐related liver disease	36 (40)	110 (47)	0.257
Metabolic dysfunction‐associated steatohepatitis	11 (12)	42 (18)	0.191
Other	16 (18)	56 (24)	0.226

*Note:* Baseline characteristics of cirrhotic patients with HCC receiving atezolizumab/bevacizumab versus cirrhotic patients without HCC are depicted as medians with interquartile ranges (IQR) or counts with frequencies (%). *p*‐values are from Wilcoxon rank sum or Fisher's chi‐squared tests.

Abbreviations: ALBI score, albumin‐bilirubin score; BMI, body mass index; INR, international normalised ratio; MELD, model for end‐stage liver disease; suPAR, soluble urokinase plasminogen activator receptor.

### 3.2 SuPAR Concentrations Are Elevated in Cirrhosis Independent of HCC Characteristics

**FIGURE 1:**
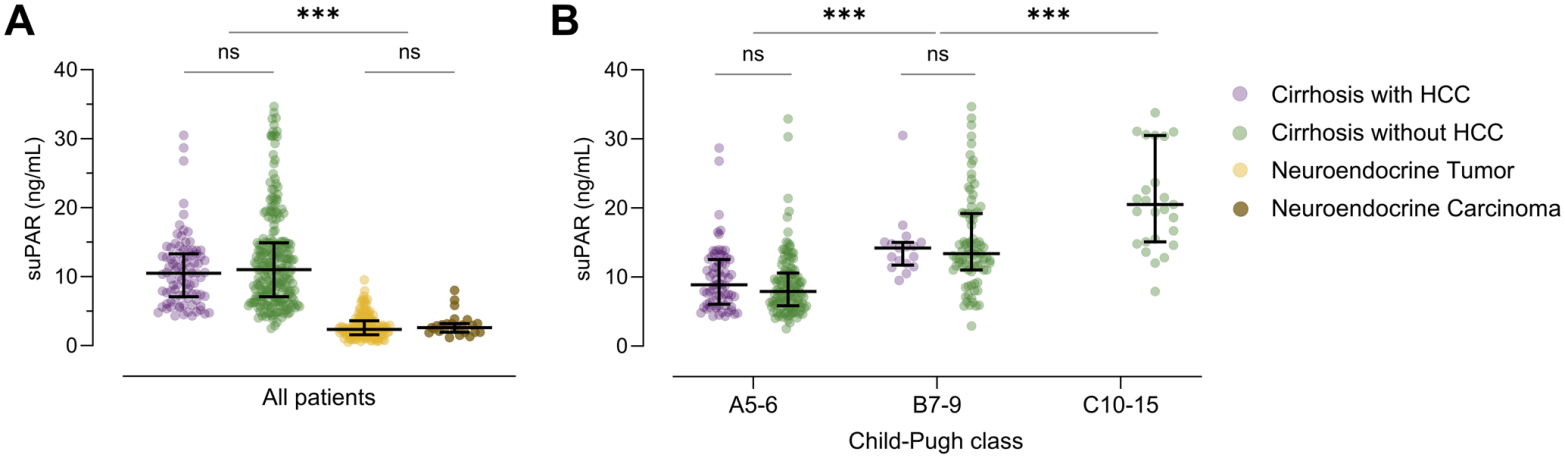
Baseline suPAR concentrations. (A) suPAR concentrations are significantly higher in cirrhotic patients with (*n* = 90) and without HCC (*n* = 235) compared to patients with neuroendocrine tumour (*n* = 132) or neuroendocrine carcinoma (*n* = 25). (B) suPAR levels show a significant stepwise increase according to Child‐Pugh class independent of the presence of HCC. Data are expressed as medians with IQR. *p*‐values are from a non‐parametric signed‐rank test accounting for between and within group differences. Ns, non‐significant; ***p < 0.001.

The median suPAR levels in patients with liver cirrhosis with vs. without HCC were 10.5 ng/mL (95% CI, 7.1–13.3) versus 11.0 ng/mL (95% CI, 7.1–14.9, *p* = 0.130). In both cohorts, median suPAR concentrations increased according to the Child‐Pugh class. Within the Child‐Pugh A and B subgroup, median suPAR levels were similar in patients with versus without HCC (Figure 1). In contrast, non‐cirrhotic cancer patients exhibited significantly lower median suPAR levels (*p* < 0.001, Figure 1). In patients with NET and NEC, suPAR concentrations were 2.4 ng/mL (95% CI, 1.6–3.6) and 2.6 ng/mL (95% CI, 2.0–3.2), respectively (*p* = 0.345).

In patients with liver cirrhosis with and without HCC, a strong correlation was observed between suPAR concentrations and laboratory parameters of liver function, for example, Model for End‐Stage Liver Disease (MELD) score (*r*_S_ = 0.493, *p* < 0.001 and 0.664, *p* < 0.001) and albumin–bilirubin (ALBI) score (*r*_S_ = −0.673, *p* < 0.001 and − 0.668, *p* < 0.001), as well as with clinical features of portal hypertension (oesophageal varices, ascites and hepatic encephalopathy, *p* < 0.005 in each case; Figure S1). In the HCC cohort, suPAR was significantly associated with ECOG performance status (*r*_S_ = 0.438, *p* = 0.001) and the immune parameters C‐reactive protein (CRP) (*r*_S_ = 0.321, *p* = 0.002) and relative lymphocyte count (*r*_S_ = −0.359, *p* = 0.011). Interestingly, no correlation was found with tumour‐specific characteristics (such as BCLC stage, macrovascular infiltration, extrahepatic spread and number of intrahepatic nodules).

Patients with alcohol‐associated liver disease (ALD) exhibited significantly higher suPAR levels compared to all other etiologies of liver cirrhosis. This was consistent for patients with HCC (11.9 ng/mL [95% CI, 8.8–13.8] vs. 9.1 ng/mL [95% CI, 5.9–12.9], *p* = 0.031), and patients without HCC (12.6 ng/mL [95% CI, 8.5–19.2] vs. 9.7 ng/mL [95% CI, 6.6–13.9], *p* = 0.004). In the HCC cohort, this might be partially explained by more advanced liver cirrhosis in patients with alcohol‐related aetiology (MELD score: 8.7 [95% CI, 7.5–10.9] vs. 7.9 [95% CI, 6.6–9.3], *p* = 0.023), but no such differences in liver function could be observed in patients without HCC. Of note, for both cirrhotic groups with and without HCC, no correlation of suPAR levels with age (*r*_S_ = 0.021, *p* = 0.842 and 0.023, *p* = 0.727) or creatinine values (*r*_S_ = 0.108, *p* = 0.413 and 0.155, *p* = 0.111) was detected.

### 3.3 SuPAR Levels Predict the Deterioration of Liver Function in Patients Receiving Atezolizumab/Bevacizumab

**FIGURE 2:**
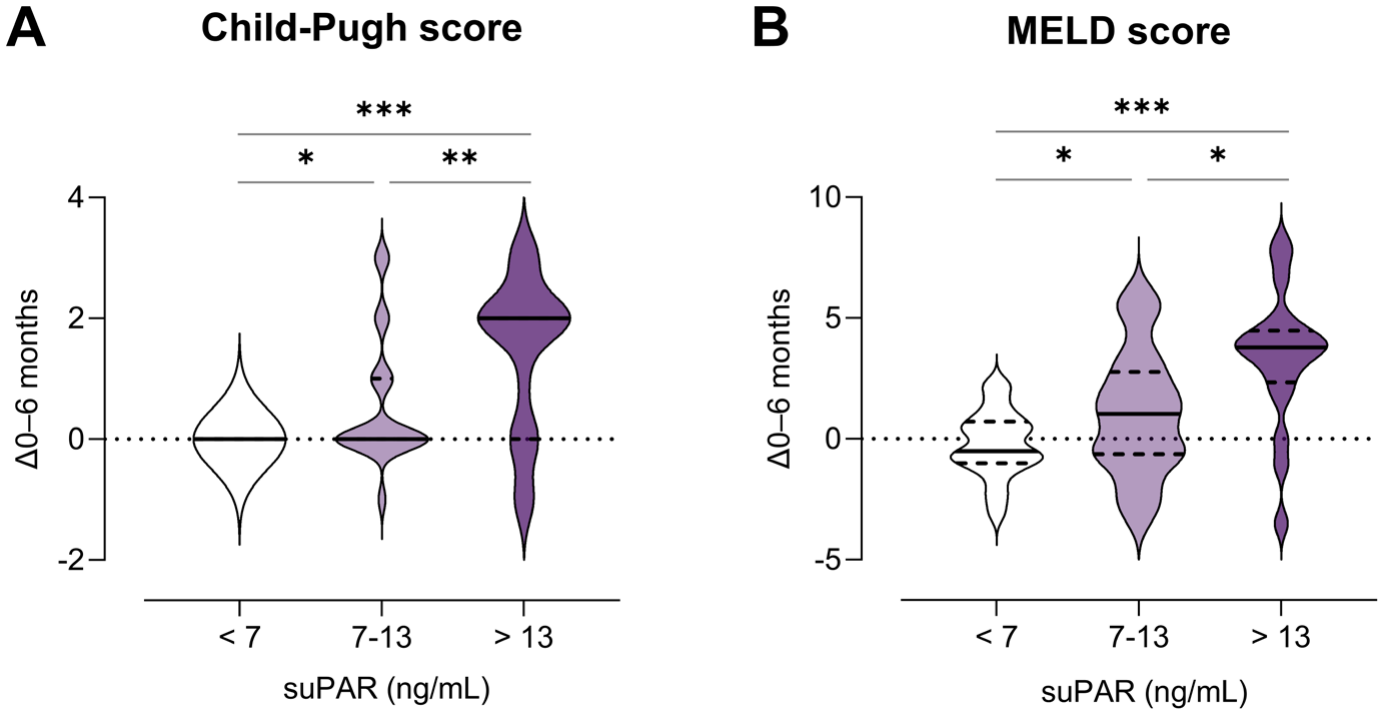
Changes in Child‐Pugh score and MELD score according to suPAR risk groups in cirrhotic patients with HCC. Depicted are absolute changes of Child‐Pugh score (A) and MELD score (B) after 6 months of therapy, risk‐stratified by quartiles of pre‐treatment suPAR levels (Q1, Q2 + 3, Q4). Data are expressed as medians with IQR. *p*‐values are from a non‐parametric signed‐rank test accounting for between‐ and within‐group differences. *p < 0.05, **p < 0.01, ***p < 0.001. MELD, model for end‐stage liver disease; suPAR, soluble urokinase plasminogen activator receptor.

In 90 HCC patients who received ICI‐based therapy, suPAR was determined before the start of therapy. SuPAR levels at baseline were significantly associated with absolute changes in the Child‐Pugh score (*r*_S_ = 0.394, *p* < 0.001), MELD score (*r*_S_ = 0.379, *p* < 0.001) and ALBI score (*r*_S_ = 0.363, *p* = 0.014) within 6 months of follow‐up. After 6 months of treatment with atezolizumab/bevacizumab, the median Child‐Pugh score increased by 2 points (95% CI, 0–2) in patients with suPAR levels in the upper quartile (Q4, suPAR > 13 ng/mL), while it remained unchanged (95% CI, 0–0) in patients with suPAR levels in the lower quartile (Q1, suPAR < 7 ng/mL; *p* < 0.001). The median MELD score increased by 38% (95% CI, 21–53) in patients from suPAR Q4, and decreased by 6% (95% CI, −12 to 12) in patients from suPAR Q1 (*p* < 0.001, Figure 2) after 6 months of ICI‐based therapy.

**FIGURE 3:**
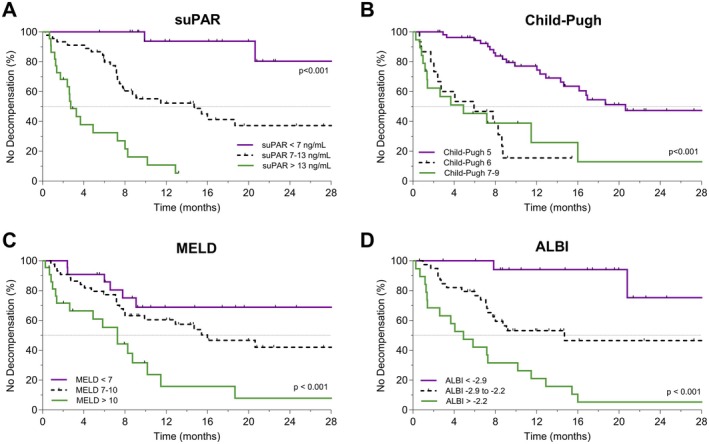
Kaplan–Meier analysis of time without hepatic decompensation in cirrhotic patients with HCC. Depicted are Kaplan–Meier estimates of time without hepatic decompensation, risk‐stratified by quartiles (Q1, Q2 + 3, Q4) of pre‐treatment suPAR levels (A), Child‐Pugh score (B), MELD score (C) and ALBI score (D) in cirrhotic patients with HCC receiving atezolizumab/bevacizumab. Tick marks indicate censored data. *p*‐values from log‐rank test are given. ALBI score, albumin‐bilirubin score; MELD, model for end‐stage liver disease; suPAR, soluble urokinase plasminogen activator receptor.

During a median follow‐up of 12.2 months (95% CI, 8.6–19.7), grade 3 or 4 serious adverse events in patients with HCC receiving atezolizumab/bevacizumab were reported in 65 patients (72%). The most common serious complication was hepatic decompensation, accounting for 51%. Patients with higher suPAR levels showed a significantly shorter time without hepatic decompensation (*p* < 0.001 using log‐rank test, Figure 3A). Kaplan–Meier estimated median time without hepatic decompensation was 28.9 months [IQR, 12.4–45.4] vs. 2.7 months [IQR, 1.8–3.7] in patients with suPAR below or above the 75th percentile. Univariate HR for time to hepatic decompensation was 6.3 (95% CI, 3.3–11.9; *p* < 0.001). Child‐Pugh, MELD, ALBI score and CRP were also significantly associated with the time without hepatic decompensation (Figure 3B–D, Figure S2B). In multivariable stepwise backwards COX‐regression, suPAR and ALBI score remained as independent predictors of time to hepatic decompensation (*p* < 0.001 in each case; Table S3).

Quantifying discriminatory accuracy by time‐dependent ROC analysis, pre‐treatment suPAR levels and ALBI score had the highest AUC values for hepatic decompensation within 6, 12 and 18 months of atezolizumab/bevacizumab treatment (0.85–0.92, Table S4). MELD score, CRP, relative lymphocyte count as well as neutrophile‐to‐lymphocyte ratio showed time‐dependent AUC values below 0.73 during the whole follow‐up period (Table S4).

### 3.4 High suPAR Levels Indicate Poor Overall Survival in Cirrhotic Patients With and Without HCC

**FIGURE 4:**
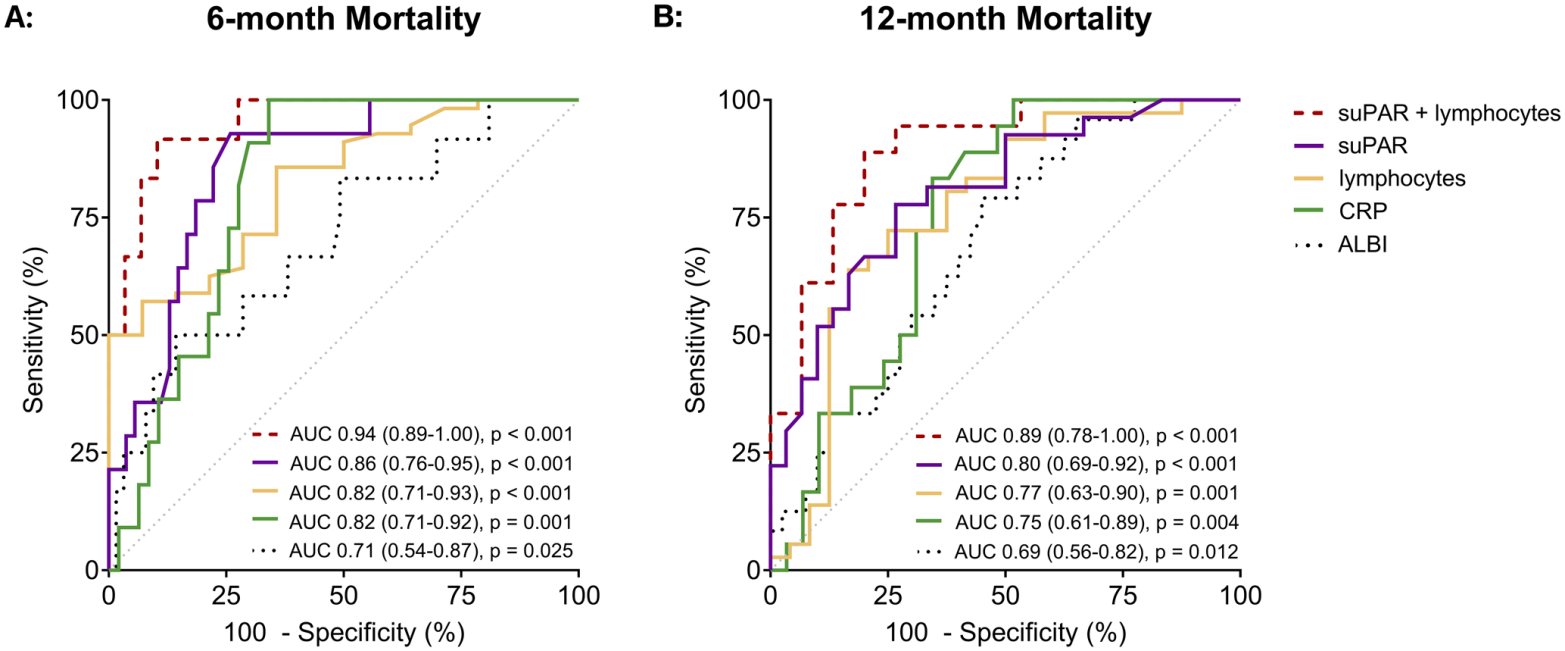
Prognostic performance for 6‐ and 12‐month mortality. Depicted are ROCs and AUCs with 95% confidence intervals (95% CI) for the prediction of 6‐ (A) and 12‐month mortality (B) in cirrhotic patients with HCC. The combination of suPAR levels and relative lymphocyte counts was performed by logistic regression. ALBI score, albumin‐bilirubin score; AUC, area under the receiver operating characteristic curve; CRP, C‐reactive protein; ROC, receiver‐operating characteristic curve; suPAR, soluble urokinase plasminogen activator receptor.

During the median follow‐up of 12.2 months in the HCC cohort, 46 patients (51%) died. SuPAR levels before initiation of atezolizumab/bevacizumab were significantly higher in patients who died within 6 months (median suPAR: 13.4 ng/mL [IQR, 11.5–16.3] vs. 8.1 ng/mL [IQR, 5.6–10.5], *p* < 0.001; Figure S3). The AUC for predicting 6‐month mortality was high for pre‐treatment suPAR levels (0.86 [95% CI, 0.76–0.95], *p* < 0.001; Figure 4).

The second most powerful predictor of 6‐month mortality was the relative lymphocyte count prior to immunotherapy initiation (AUC 0.82 [95% CI, 0.71–0.93], *p* < 0.001). The discriminative power for 6‐month mortality could be further improved by combining suPAR values and relative lymphocyte counts by logistic regression (AUC 0.94 [95% CI, 0.89–1.00], *p* < 0.001). The AUC remained high for the prediction of 12‐month mortality (Figure 4). The results were confirmed by time‐dependent ROC analysis over a follow‐up period of 18 months (Table S5).

Kaplan–Meier estimated OS was 5.7 months (95% CI, 0.8–12.6) versus 20.4 months (95% CI 12.6–28.3) in HCC patients receiving atezolizumab/bevacizumab with suPAR levels above vs. below the 75th percentile (univariate HR 4.4 [95% CI, 2.3–8.5], *p* < 0.001). Using a multivariable Cox regression model with stepwise backward selection, suPAR remained the only independent laboratory parameter significantly associated with OS (stratified HR: 1.12 [95% CI, 1.06–1.18], *p* < 0.001), besides the baseline characteristics ECOG performance status and age (Table 2).

**TABLE 2:** Uni‐ and multivariate Cox‐regression analyses for overall survival in cirrhotic patients with HCC.

Parameter	Univariate regression models		Multivariate regression models	
	*p*	Hazard ratio (95% CI)	*p*	Hazard ratio (95% CI)
Male	0.285	0.64 (0.28–1.45)		
Age	0.003	1.05 (1.02–1.09)	0.007	1.07 (1.02–1.12)
BMI	0.892	1.00 (0.93–1.07)		
ECOG performance status score				
1	0.021	2.27 (1.13–4.56)	0.665	1.24 (0.47–3.24)
2	< 0.001	6.07 (2.69–13.72)	0.006	4.31 (1.51–12.29)
Other characteristics of liver cirrhosis				
Oesophageal varices	0.006	2.37 (1.29–4.37)	0.686	0.82 (0.31–9.14)
Ascites	0.001	2.67 (1.49–4.87)	0.081	2.13 (0.91–4.99)
Laboratory parameters				
suPAR	< 0.001	1.11 (1.07–1.16)	< 0.001	1.12 (1.06–1.18)
Child‐Pugh score	< 0.001	1.55 (1.26–1.91)	0.751	0.90 (0.50–1.60)
MELD score	< 0.001	1.18 (1.09–1.27)	0.426	0.94 (0.80–1.10)
Creatinine	< 0.001	2.76 (1.58–4.8)	0.215	1.64 (0.75–3.57)
ALBI score	0.005	2.15 (1.25–3.67)	0.741	1.26 (0.33–4.85)
CRP	0.747	1.00 (0.99–1.01)		
Aetiology of liver cirrhosis				
Hepatitis B	0.365	0.72 (0.36–1.46)		
Hepatitis C	0.333	0.67 (0.30–1.51)		
Alcohol‐related liver disease	0.731	1.11 (0.62–1.98)		
Metabolic dysfunction‐associated steatohepatitis	0.167	1.85 (0.77–4.43)		
Characteristics of hepatocellular carcinoma				
BCLC stage B	0.679	0.88 (0.48–1.62)		
Macrovascular invasion	0.745	0.91 (0.50–1.65)		
Extrahepatic spread	0.369	1.32 (0.72–2.34)		
Relevant comorbidities				
Chronic kidney disease	0.001	2.87 (1.55–5.32)	0.648	1.27 (0.46–3.52)
Autoimmune disease	0.395	0.54 (0.13–2.23)		
Cardiac disease	0.015	2.15 (1.16–3.99)	0.494	1.31 (0.60–2.86)

Abbreviations: ALBI score, albumin‐bilirubin score; BCLC, Barcelona Clinic liver cancer stage; BMI, body mass index; CRP, C‐reactive protein; ECOG, Eastern Cooperative Oncology Group; MELD, model for end‐stage liver disease; suPAR, soluble urokinase plasminogen activator receptor.

In the control group of cirrhotic patients without HCC, the median OS also decreased significantly with increasing suPAR levels. According to the Kaplan–Meier analysis, suPAR showed a similar prognostic accuracy regarding OS stratification compared with the MELD or ALBI score (Figure S4). However, the ALBI score had higher AUC values than suPAR and the MELD score for predicting mortality within 6, 12 and 18 months (Table S6). In a multivariable Cox‐regression model using stepwise backward selection, ALBI and the Child‐Pugh score remained as independent predictors of OS (*p* = 0.005 and 0.013, Table S7).

Both in cirrhotic patients with and without HCC, the OS deteriorated almost linearly with increasing suPAR levels. The HR was consistent over the entire spectrum of suPAR levels (Figure S5). The prognostic properties of suPAR were found to be independent of the aetiology of liver cirrhosis.

In the non‐cirrhotic cancer control cohort, suPAR levels were also significantly associated with mortality according to the Cox‐proportional hazards model (*p* = 0.025). However, in terms of OS, there were no significant differences for all patients with NEN or patients with NET versus NEC when stratified by suPAR quartiles (Figure S6).

### 3.5 SuPAR Levels Are Not Associated With the Response to Atezolizumab/Bevacizumab

Among all patients with HCC treated with atezolizumab/bevacizumab, 28 (36%) achieved objective treatment response. SuPAR values were evenly distributed between patients with versus without objective treatment response (8.5 ng/mL [95% CI, 6.0–11.9] vs. 11.4 ng/mL [95% CI, 7.2–13.5], *p* = 0.099, respectively). Furthermore, no significant association of suPAR and primary disease progression could be detected (*p* = 0.754 using a univariate Cox‐regression model).

### 3.6 Hepatic Decompensation Indicates Poor Overall Survival Independent of Treatment Response

**FIGURE 5:**
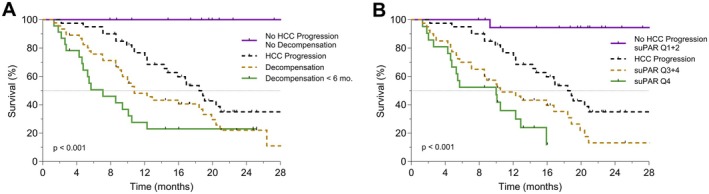
Hepatic decompensation and suPAR levels predict overall survival. Depicted are Kaplan–Meier estimates of overall survival according to HCC progression plus hepatic decompensation (A) and HCC progression plus suPAR quartiles (B). Tick marks indicate censored data. *p*‐values from log‐rank test are given. SuPAR, soluble urokinase plasminogen activator receptor.

During follow‐up, 41 patients (46%) experienced HCC progression and 18 (20%) developed neither progression nor hepatic decompensation (Figure 5A). Hepatic decompensation and HCC progression were independent predictors of death according to time‐dependent multivariate COX regression analysis (stratified HRs: 5.7 [95% CI, 3.0–11.1], *p* < 0.001 and 2.5 [95% CI, 1.2–5.2], *p* = 0.013, respectively). OS was shorter in patients suffering hepatic decompensation but no tumour progression, when compared to patients suffering tumour progression but no hepatic decompensation (Figure S7). The individual discriminatory power could be further improved by combining the information from treatment response and hepatic decompensation (Figure S8A–F). Moreover, quartiles of baseline suPAR levels were able to stratify OS to the same extent as the presence of hepatic decompensation within 6 and 12 months (Figure 5B and Figure S8G–I).

### 3.7 Longitudinal suPAR Measurements in Cirrhotic Patients With HCC

Serial suPAR measurements were available in 62% of HCC patients receiving atezolizumab/bevacizumab. Median deviation from baseline was 20% (95% CI, 14–34) and median deviation from mean was 13% (95% CI, 9–20). In patients with versus without hepatic decompensation during the first 6 months of treatment, absolute change from baseline levels was significantly lower (1.5 ng/mL [95% CI, 0.8–2.4] vs. 4.2 ng/mL [95% CI, 2.0–5.8], *p* = 0.001). The same observation was made for 6‐month mortality (1.5 ng/mL [95% CI, 0.9–2.4] vs. 4.7 ng/mL [95% CI, 3.9–7.7], *p* = 0.001). The maximum change in suPAR levels during the first 6 months of treatment (∆suPAR 0–6 Max.) was significantly associated with the time to decompensation and OS (Figure S9A,B). AUCs from ROC analysis for predicting 6‐month mortality and 6‐month decompensation were similar for ∆suPAR 0–6 Max. and for baseline suPAR concentrations (OS: 0.82 [95% CI, 0.58–1.00], *p* = 0.009 vs. 0.86 [95% CI, 0.76–0.95], *p* < 0.001; decompensation: 0.85 [95% CI, 0.72–0.98], *p* = 0.001 vs. 0.84 [95% CI, 0.75–0.93], *p* < 0.001). Combining pre‐treatment suPAR levels with maximum 6‐month changes by logistic regression further increased the prognostic accuracy (Figure S9C,D).

### 3.8 Child‐Pugh Class Subgroup Analysis in Cirrhotic Patients With HCC

Within the HCC cohort receiving atezolizumab/bevacizumab, Child‐Pugh A specific suPAR quartiles were able to further stratify patients according to their mortality risk and risk of hepatic decompensation (Figure S10A,B). Patients with Child‐Pugh A cirrhosis and a suPAR value in Q4 had a similar median survival and time to hepatic decompensation as patients with Child‐Pugh B cirrhosis independent of their suPAR level (OS: 9.3 months [95% CI, 6.1–12.5] vs. 12.3 months [95% CI, 6.7–17.9], *p* = 0.499; Figure S10C,D).

**FIGURE 6:**
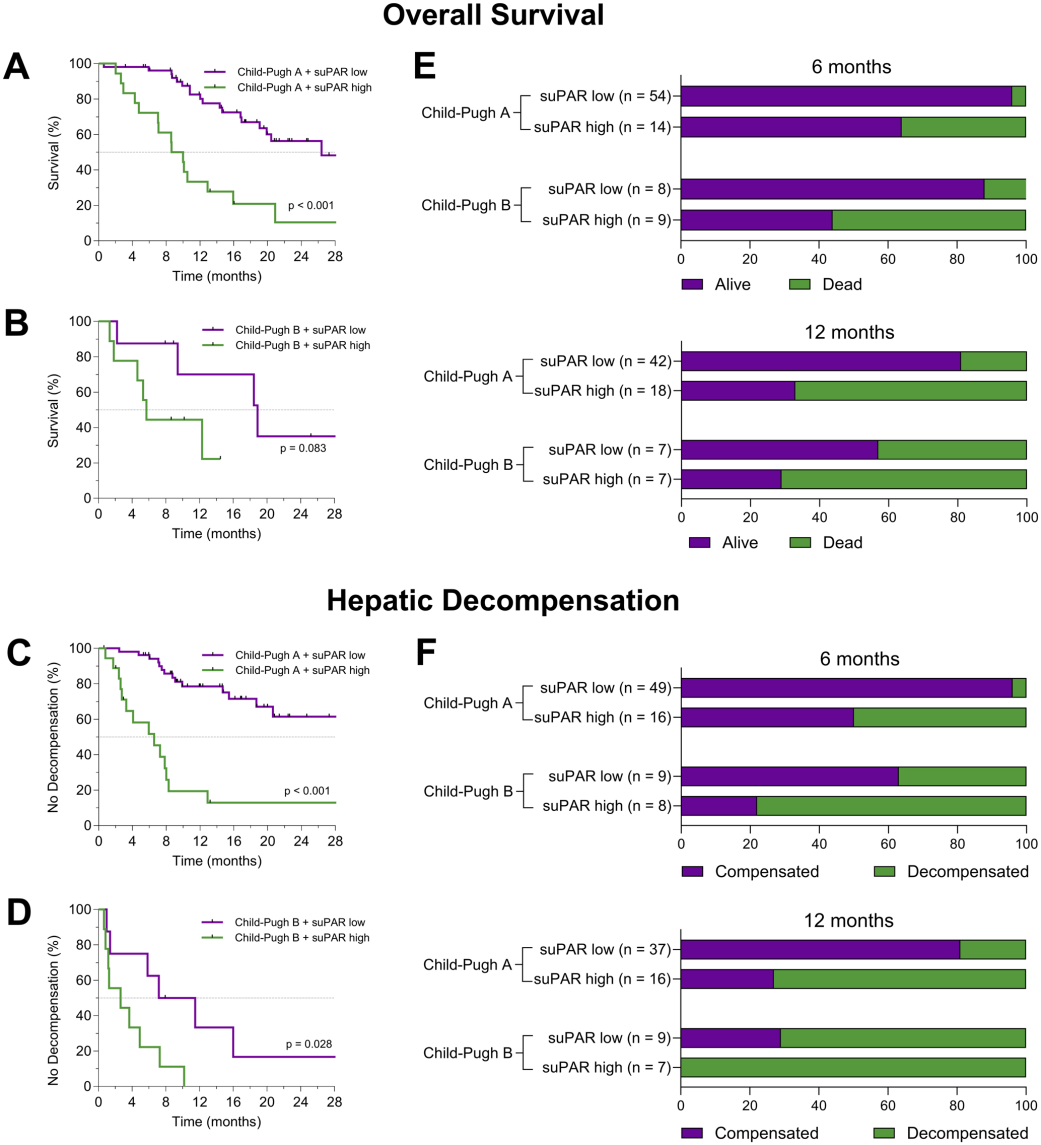
SuPAR identifies patients with poor outcomes within the Child‐Pugh classes in cirrhotic patients with HCC. (A–D) Depicted are Kaplan–Meier estimates of overall survival and time without hepatic decompensation in patients with Child‐Pugh class A cirrhosis (A, C) and patients with Child‐Pugh class B cirrhosis (B, D). Patients were stratified for low versus high baseline suPAR concentrations (< 12 ng/mL vs. ≥ 12 ng/mL), with 12 ng/mL corresponding to the upper quartile within the Child‐Pugh A group. (E, F) Number of deaths (E) and hepatic decompensations (F) after 6 and 12 months stratified by Child‐Pugh class and low versus high baseline suPAR levels.

Most interestingly, Child Pugh classes A and B could each be divided into low and high suPAR subgroups (≥ vs. < 12 ng/mL) with similar survival properties. 12 ng/mL corresponds to quartile 4 within the Child‐Pugh A group. Regardless of their liver function as defined by the Child‐Pugh classification, patients with suPAR ≥ 12 ng/mL showed comparable Kaplan–Meier estimates of OS (Figure 6A,C) and time without hepatic decompensation (Figure 6D,F). ALBI grades were also able to further risk‐stratify patients in terms of hepatic decompensation within the Child Pugh A subgroup, but not significantly within the Child‐Pugh B class (Figure S11D–F). Within the Child‐Pugh subgroups, no significant prognostic properties of ALBI grades were observed for OS (Figure S11A–C).

## 4 Discussion

The incidence of HCC is rising, making it a major contributor to the world's cancer burden. Although the introduction of ICI‐based regimens has improved the management of HCC, response rates to systemic therapies remain poor. Moreover, advanced liver cirrhosis often precludes patients from treatment. In contrast to other tumour entities, OS in HCC patients is determined not only by the oncological course but to a large extent also by the degree of liver function impairment. Early deterioration of liver function during systemic therapy is often irreversible and represents the most common cause of permanent treatment interruption [14, 41]. Pre‐treatment identification of appropriate candidates for systemic therapies is crucial to optimise treatment efficacy, avoid hepatic decompensation and reduce treatment expenses. Thus, non‐invasive and easily accessible biomarkers are required for a personalised oncological decision‐making.

To the best of our knowledge, this is the first study investigating suPAR as a potential novel biomarker in patients with advanced HCC in the context of ICI therapy. In order to differentiate the influence of liver cirrhosis and HCC on circulating suPAR levels, patients with liver cirrhosis without HCC, and non‐cirrhotic and treatment‐naïve cancer patients with GEP‐NEN were evaluated as control cohorts. We demonstrated that suPAR is significantly elevated in patients with liver cirrhosis independent of the presence of HCC compared to patients with GEP‐NEN. A strong association with liver function and systemic inflammation, but not with HCC characteristics, could be identified. This is in line with previous data showing increased suPAR levels in liver diseases [15, 42].

Whereas suPAR was associated with response to immunotherapy in other malignancies [25, 43], we could not observe a correlation with treatment response nor HCC characteristics. However, in our HCC cohort suPAR‐quartiles were able to predict hepatic decompensation and biochemical deterioration of liver function. Kaplan–Meier estimated median time without hepatic decompensation was 25.5 months [IQR, 22.0–29.0] versus 9.0 months [IQR, 6.3–11.7] in patients with a suPAR below vs. above the median of 10.5 ng/mL. In patients with HCC, hepatic decompensation indicated poor OS independent of treatment response, which is consistent with recently published data in HCC patients with Child‐Pugh A cirrhosis receiving atezolizumab/bevacizumab treatment [14]. Our study confirmed a higher mortality risk for patients with hepatic decompensation than for patients who progressed without decompensation. Remarkably, baseline suPAR levels were able to predict OS to the same extent as hepatic decompensation. SuPAR was the most accurate independent predictor of OS according to multivariate COX regression and time‐dependent ROC analysis. In contrast, MELD and ALBI score were superior to suPAR in the cirrhotic control cohort without HCC.

Treatment decisions for patients with HCC and impaired liver function are particularly challenging. Prospective evidence of ICI use in Child‐Pugh B patients is largely missing. Retrospective analyses show conflicting results with regard to the safety and efficacy of ICI in this population [8, 10, 11, 12, 13, 44].

Selection whether to use ICI in patients with impaired liver function is approximated with the Child‐Pugh or ALBI score, although no clear cut‐off values have yet been established. Meta‐analysis concludes that although OS and PFS are lower, a proportion of Child‐Pugh B patients might benefit from ICI therapy [8, 11, 44]. The poor overall prognosis in this group mainly results from the competing effects of cirrhosis‐related complications under systemic therapy.

In contrast to conventional liver function scores, suPAR additionally reflects the level of systemic inflammation. Since the degree of systemic inflammation indicates the risk of liver function deterioration and developing ACLF [33, 34, 35], suPAR might identify patients with a lower capacity to withstand the inflammatory ‘overload’ which is provoked by ICI. In our study, suPAR was able to stratify the risk of hepatic decompensation within distinct Child‐Pugh classes. For example, patients with advanced cirrhosis Child‐Pugh B and low suPAR levels seemed to benefit from immunotherapy to the same extent as patients with preserved liver function Child‐Pugh A.

In recent publications, suPAR has been described as a marker with minimal circadian fluctuation and low within‐person variability [17]. In acute complications, such as pneumonia or trauma, only marginal and delayed changes were described in suPAR concentrations [45, 46, 47]. In line, we observed stable within‐patient suPAR levels over a 6‐month period. Nevertheless, patients with rising suPAR concentrations during ICI therapy, as measured by the absolute changes within the first 6 months of treatment, exhibited a shorter time to hepatic decompensation and shorter OS. Whereas the pre‐treatment suPAR level has a high prognostic value, the combination with the changes from baseline might further increase its prognostic accuracy. Still, a one‐time determination of suPAR prior to immunotherapy initiation appears warrantable as a prognostic parameter for precise risk stratification and treatment decision making.

We acknowledge important limitations of our study, most of which cannot be avoided due to the retrospective study design. First, it was conducted at three national tertiary care centers with a relatively small number of patients. Second, the real‐life setting of our study is associated with a lack of standardisation in clinical practice, including eligibility and treatment algorithm. Third, the lack of randomization might have introduced several natures of biases, including selection bias, information bias and observation bias. Fourth, the cohort sizes, especially for Child‐Pugh B cirrhosis, were too small for detailed assessment. Fifth, longitudinal suPAR measurements were only available for 62% of cirrhotic patients with HCC. Sixth, we were unable to provide information on the molecular mechanism being responsible for the better prognostic performance in patients with HCC receiving immunotherapy compared to cirrhotic patients without HCC. Nonetheless, our findings raise intriguing questions about the potential clinical utility of suPAR as a prognostic biomarker in patients with HCC treated with atezolizumab and bevacizumab.

In summary, our data provide strong evidence that baseline suPAR concentrations represent a novel prognostic marker in patients with HCC treated with atezolizumab and bevacizumab. SuPAR assessment could serve as a non‐invasive tool for patient stratification, identifying subgroups beyond common scores of liver function (like Child Pugh, MELD or ALBI). Especially in view of treatment regimens using double immune checkpoint inhibition (e.g., tremelimumab plus durvalumab or ipilimumab plus nivolumab) or immune response boosting through tumour antigen release (e.g., cancer vaccines or locoregional therapies combined with systemic ICI), suPAR may serve as a valuable biomarker.

Determination of suPAR levels prior to systemic therapy initiation has the potential to guide clinical decision making. It may identify patients who benefit from immunotherapy in terms of hepatologic tolerability and OS, and those with a lower capacity to withstand the immunological challenges induced by immunotherapy. Latter may need alternative or additional treatment approaches, as well as close hepatologic monitoring. SuPAR guided treatment decisions might be particularly valuable in patients with advanced cirrhosis Child‐Pugh B.

Our findings provide evidence for further confirmation in longitudinal clinical trials using independent cohorts. Our results might open the door for a potential clinical use of circulating suPAR as a non‐invasive risk prediction tool in this challenging clinical setting.

## Author Contributions

Fabian Artusa: Conceptualization (equal); data curation (equal); formal analysis (equal); methodology (equal); visualisation (equal); writing – original draft (equal). Sven Lamatsch, Minh Duc Phan and Burcin Özdirik: Data curation (equal); validation (equal). Hilmar Berger: Formal analysis (equal); methodology (equal). Mara Egerer: Data curation (equal); writing – review and editing (equal). Jana Knorr‐Klocke: Data curation (equal); methodology (equal). Janett Fischer, Rhea Veelken, Florian van Bömmel and Thomas Berg: Investigation (equal); validation (equal). Kai Kappert, Rudolf Tauber and Cornelius Engelmann: Methodology (equal); writing – review and editing (equal). Tobias Puengel: Investigation (equal), writing – review and editing. Münevver Demir: Data curation (equal); methodology (equal); writing – review and editing (equal). Frank Tacke: Investigation (equal); supervision (equal); writing – review and editing (equal). Raphael Mohr: Conceptualization (equal); data curation (equal); investigation (equal); project administration (equal); supervision (equal); writing – review and editing (equal).

## Ethics Statement

The study was conducted according to the guidelines of the Declaration of Helsinki and approved by the Institutional Review Board of Charité–Universitätsmedizin Berlin (EA2/091/19).

## Conflicts of Interest

The authors declare no conflicts of interest.

## Supporting information


Appendix S1.

## Data Availability

The data presented in this study are available on request from the corresponding author.
