# Supplementary material for: Soluble Urokinase Plasminogen Activator Receptor Predicts Survival and Hepatic Decompensation in Advanced Hepatocellular Carcinoma
Source: Liver Int. 2025 May 2;45(6):e70121. doi: 10.1111/liv.70121 (PMC12046945; doi:10.1111/liv.70121)
Supplement: Supplementary file 1 — Appendix S1. [file LIV-45-0-s001.docx]

**Soluble Urokinase Plasminogen Activator Receptor Predicts
Survival and Hepatic Decompensation in Advanced Hepatocellular Carcinoma**

**SUPPORTING INFORMATION**

Fabian Artusa, Sven Lamatsch, Minh Duc Phan, Burcin Özdirik, Hilmar Berger, Mara Egerer, Jana Knorr-Klocke, Janett Fischer, Rhea Veelken, Florian van Bömmel, Thomas Berg, Kai Kappert, Rudolf Tauber, Tobias Puengel, Cornelius Engelmann, Münevver Demir, Frank Tacke, Raphael Mohr

[Table S1. Baseline patient characteristics of cirrhotic patients with HCC receiving atezolizumab / bevacizumab 3](#_Toc188476779)

[Table S2. Baseline patient characteristics of non-cirrhotic cancer patients with neuroendocrine tumors vs. neuroendocrine carcinomas 4](#_Toc188476780)

[Figure S1. SuPAR concentrations in cirrhotic patients with and without HCC according to liver function. 5](#_Toc188476781)

[Figure S2. Kaplan–Meier analysis of overall survival and time without hepatic decompensation in cirrhotic patients with HCC according to CRP risk groups. 5](#_Toc188476782)

[Table S3. Uni- and multivariate Cox-regression analyses for hepatic decompensation in cirrhotic patients with HCC. 6](#_Toc188476783)

[Table S4. Time-dependent AUCs for hepatic decompensation in cirrhotic patients with HCC. 7](#_Toc188476784)

[Figure S3. SuPAR levels correlate with short- and long-term mortality in cirrhotic patients with HCC. 7](#_Toc188476785)

[Table S5. Time-dependent AUCs for overall survival in cirrhotic patients with HCC. 8](#_Toc188476786)

[Figure S4. Kaplan–Meier analysis of overall survival in cirrhotic patients with and without HCC. 9](#_Toc188476787)

[Table S6. Time-dependent AUCs for overall survival in cirrhotic patients without HCC. 10](#_Toc188476788)

[Table S7. Uni- and multivariate Cox-regression analyses for overall survival in cirrhotic patients without HCC. 10](#_Toc188476789)

[Figure S5. Mortality risk according to different suPAR cut-off values. 11](#_Toc188476790)

[Figure S6. Kaplan–Meier analysis of overall survival in patients with gastroenteropancreatic neuroendocrine neoplasia. 11](#_Toc188476791)

[Figure S7. Kaplan–Meier analysis of overall survival according to HCC progression and hepatic decompensation in cirrhotic patients with HCC. 12](#_Toc188476792)

[Figure S8. Kaplan–Meier analysis of overall survival according to treatment response, hepatic decompensation and suPAR levels in cirrhotic patients with HCC. 12](#_Toc188476793)

[Figure S9. Kaplan–Meier analysis of overall survival and time without hepatic decompensation according to changes in suPAR levels in cirrhotic patients with HCC. 13](#_Toc188476794)

[Figure S10. Kaplan–Meier analysis of overall survival and time without hepatic decompensation in patients with HCC and Child-Pugh A cirrhosis. 14](#_Toc188476795)

[Figure S11. Child-Pugh class subgroup analysis according to ALBI grades in cirrhotic patients with HCC. 15](#_Toc188476796)

## Table S1. Baseline patient characteristics of cirrhotic patients with HCC receiving atezolizumab / bevacizumab

|  | Atezolizumab / Bevacizumab  (n=90) |
| --- | --- |
| Median age, median (IQR)—year | 67 (60-72) |
| Male sex, n (%) | 77 (86) |
| BMI median (IQR)—kg/m^2^ | 27 (24-30) |
| ECOG performance status score, n (%) |  |
| 0 | 61 (68) |
| 1 | 19 (21) |
| 2 | 10 (11) |
| Child–Pugh classification, n (%) |  |
| A5-6 | 71 (79) |
| B7-9 | 17 (19) |
| C10 | 2 (2) |
| Other characteristics of liver cirrhosis, n (%) |  |
| Esophageal varices | 48 (53) |
| Treated at baseline | 25 (28) |
| Previous variceal bleeding | 16 (18) |
| Ascites at baseline | 30 (33) |
| Prior hydropic decompensation | 16 (18) |
| Prior hepatic encephalopathy | 8 (9) |
| Relevant comorbidities, no. (%) |  |
| Chronic Kidney Disease | 20 (22) |
| Autoimmune disease | 5 (6) |
| Cardiac disease | 51 (57) |
| Laboratory parameters |  |
| MELD score, median (IQR) | 8 (7-10) |
| ALBI score, median (IQR) | -2.6 (-2.9 to -2.2) |
| Thrombocytes, median (IQR)—/nL | 145 (109-225) |
| Alpha-fetoprotein, median (IQR)—ng/mL | 33 (8-994) |
| Etiology of liver cirrhosis, n (%) |  |
| Hepatitis B | 17 (19) |
| Hepatitis C | 10 (11) |
| Alcohol-related liver disease | 36 (40) |
| Metabolic dysfunction-associated steatohepatitis | 11 (12) |
| Other | 16 (18) |
| Characteristics of hepatocellular carcinoma |  |
| Barcelona Clinic liver cancer stage, no. (%) |  |
| B | 32 (36) |
| C | 58 (64) |
| Macrovascular invasion, no. (%) | 35 (39) |
| Extrahepatic spread, no. (%) | 31 (34) |
| Prior local therapy for HCC, no. (%) | 45 (50) |

Depicted are medians with interquartile ranges (IQR) or counts with frequencies (%). p-values are from Wilcoxon rank sum or Fisher´s chi-squared tests. ALBI score, albumin-bilirubin score; BMI, body mass index; ECOG, Eastern Cooperative Oncology Group; MELD, model for end-stage liver disease; suPAR, soluble urokinase plasminogen activator receptor.

## Table S2. Baseline patient characteristics of non-cirrhotic cancer patients with neuroendocrine tumors vs. neuroendocrine carcinomas

|  | Neuroendocrine Tumor  (n=132) | Neuroendocrine Carcinoma (n=25) | p-value |
| --- | --- | --- | --- |
| Median age, median (IQR)—year | 60 (48-67) | 66 (50-71) | 0.153 |
| Survival, median (IQR)—year | 7.5 (3.6-11.8) | 4.3 (1.6-8.0) | 0.005 |
| SuPAR, median (IQR) | 2.4 (1.6-3.6) | 2.6 (2.0-3.2) | 0.345 |
| Male sex, n (%) | 63 (48) | 14 (56) | 0.520 |
| Primary tumor localization, n (%) |  |  |  |
| Pancreas | 63 (48) | 12 (48) | 1.000 |
| Gastrointestinal | 69 (52) | 13 (52) | 1.000 |
| Metastases, n (%) | 105 (80) | 21 (84) | 0.372 |
| Liver metastasis | 57 (58) | 15 (60) | 1.000 |
| Grading, n (%) |  |  |  |
| G1 | 68 (52) | 0 (0) | <0.001 |
| G2 | 54 (41) | 2 (8) | <0.001 |
| G3 | 10 (8) | 23 (92) | <0.001 |
| T-stage |  |  |  |
| T1 | 7 (7) | 0 (0) | 0.414 |
| T2 | 28 (26) | 2 (22) | 0.793 |
| T3 | 41 (38) | 3 (33) | 0.767 |
| T4 | 30 (28) | 4 (44) | 0.313 |
| Ki-67 index (%), median (IQR) | 2 (1-5) | 25 (20-50) | <0.001 |
| Creatinine, median (IQR)—mg/dL |  | 0.8 (0.7-1.0) |  |

Depicted are medians with interquartile ranges (IQR) or counts with frequencies (%). p-values are from Wilcoxon rank sum or Fisher´s chi-squared tests. SuPAR, soluble urokinase plasminogen activator receptor.

## Figure S1. SuPAR concentrations in cirrhotic patients with and without HCC according to liver function.


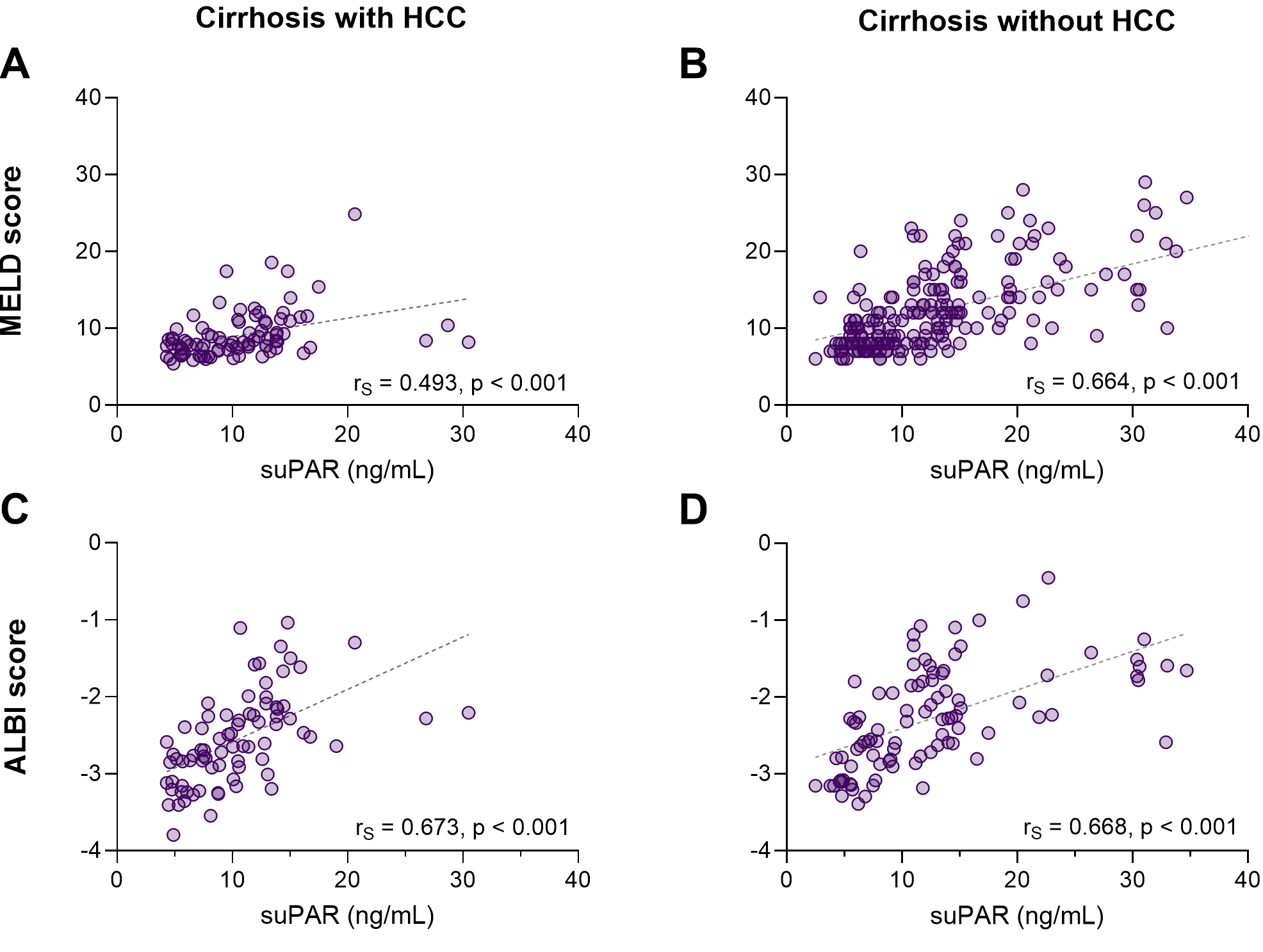
SuPAR levels significantly correlate with MELD score (A,B) and ALBI score (C,B) independent of the presence of HCC (Spearman correlation coefﬁcient). ALBI score, albumin-bilirubin score; MELD, model for end-stage liver disease; suPAR, soluble urokinase plasminogen activator receptor.

## Figure S2. Kaplan–Meier analysis of overall survival and time without hepatic decompensation in cirrhotic patients with HCC according to CRP risk groups.


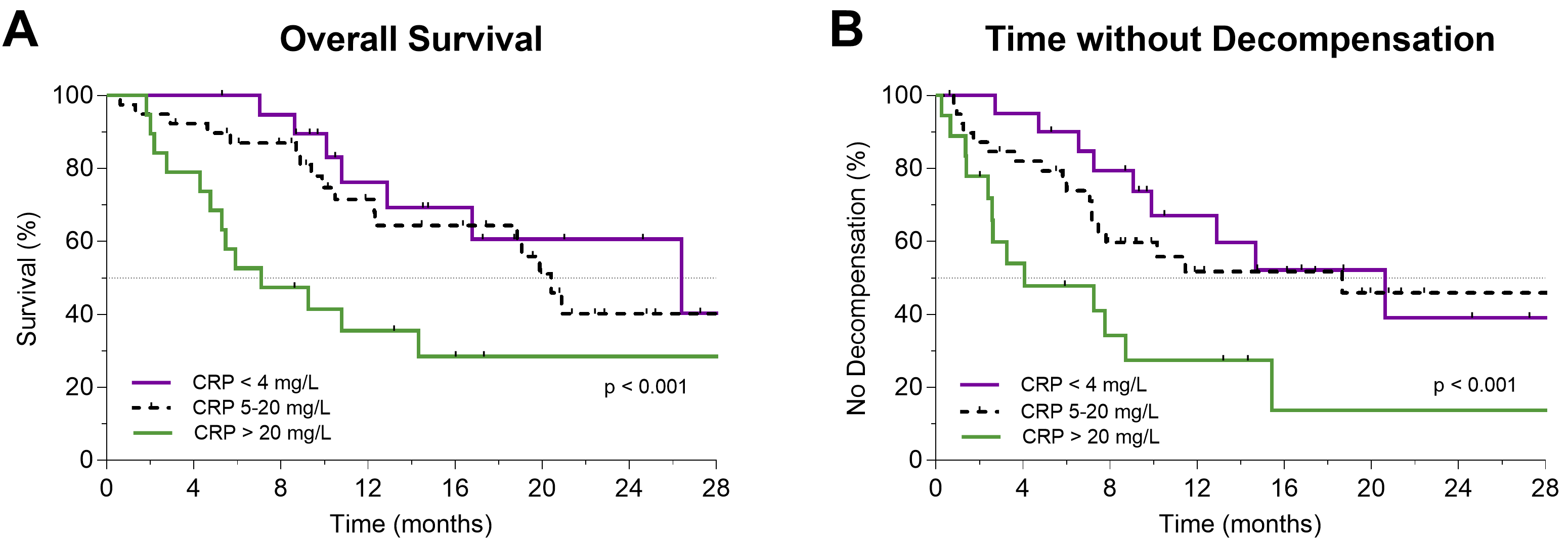
Depicted are Kaplan–Meier estimates of overall survival (A) and time without hepatic decompensation (B), risk-stratified by quartiles of CRP levels (Q1, Q2+3, Q4). Tick marks indicate censored data. p-values from log-rank test are given. CRP, C-reactive protein.

## Table S3. Uni- and multivariate Cox-regression analyses for hepatic decompensation in cirrhotic patients with HCC.

|  | *Univariate regression models* | |  | *Multivariate regression models* | |
| --- | --- | --- | --- | --- | --- |
| **Parameter** | **p-value** | **Hazard ratio (95% CI)** |  | **p-value** | **Hazard ratio (95% CI)** |
| Male | 0.877 | 0.93 (0.39-2.22) |  |  |  |
| Age | 0.665 | 0.99 (0.97-1.02) |  |  |  |
| BMI | 0.774 | 0.99 (0.93-1.06) |  |  |  |
| ECOG performance status score |  |  |  |  |  |
| 1 | 0.376 | 1.41 (0.66-2.99) |  |  |  |
| 2 | <0.001 | 6.04 (2.69-13.59) |  | 0.041 | 2.52 (1.04-6.13) |
| Other characteristics of liver cirrhosis |  |  |  |  |  |
| Esophageal varices | <0.001 | 3.84 (2.00-7.37) |  | 0.292 | 1.59 (0.67-3.78) |
| Ascites | 0.001 | 2.67 (1.49-4.79) |  | 0.692 | 0.81 (0.28-2.33) |
| Laboratory parameters |  |  |  |  |  |
| suPAR | <0.001 | 1.17 (1.12-1.23) |  | <0.001 | 1.14 (1.07-1.21) |
| Child-Pugh score | <0.001 | 2.00 (1.64-2.44) |  | 0.250 | 1.40 (0.79-2.47) |
| MELD score | <0.001 | 1.20 (1.11-1.29) |  | 0.269 | 0.92 (0.78-1.07) |
| Creatinine | 0.653 | 1.22 (0.51-2.90) |  |  |  |
| ALBI score | <0.001 | 4.76 (2.90-7.81) |  | <0.001 | 3.16 (1.81-5.52) |
| CRP | 0.719 | 1.00 (0.99-1.01) |  |  |  |
| Etiology of liver cirrhosis |  |  |  |  |  |
| Hepatitis B | 0.987 | 1.01 (0.52-1.95) |  |  |  |
| Hepatitis C | 0.995 | 1.00 (0.48-2.07) |  |  |  |
| Alcohol-related liver disease | 0.457 | 1.25 (0.70-2.23) |  |  |  |
| Metabolic dysfunction-associated steatohepatitis | 0.898 | 1.06 (0.42-2.70) |  |  |  |
| Characteristics of hepatocellular carcinoma |  |  |  |  |  |
| BCLC stage B | 0.468 | 1.24 (0.69-2.23) |  |  |  |
| Macrovascular invasion | 0.531 | 0.82 (0.45-1.51) |  |  |  |
| Extrahepatic spread | 0.981 | 0.99 (0.53-1.86) |  |  |  |
| Relevant comorbidities |  |  |  |  |  |
| Chronic kidney disease | 0.025 | 2.10 (1.10-4.02) |  | 0.532 | 1.45 (0.46-4.60) |
| Autoimmune disease | 0.154 | 0.24 (0.03-1.72) |  |  |  |
| Cardiac disease | 0.606 | 1.17 (0.65-2.08) |  |  |  |

ALBI score, albumin-bilirubin score; BCLC, Barcelona Clinic liver cancer stage; BMI, body mass index; CRP, C-reactive protein; ECOG, Eastern Cooperative Oncology Group; MELD, model for end-stage liver disease; suPAR, soluble urokinase plasminogen activator receptor.

##

## Table S4. Time-dependent AUCs for hepatic decompensation in cirrhotic patients with HCC.

| Biomarker | 6 months | 12 months | 18 months |
| --- | --- | --- | --- |
| suPAR | 0.86 (0.78-0.95) | 0.85 (0.74-0.94) | 0.92 (0.83-1.00) |
| ALBI score | 0.85 (0.75-0.94) | 0.85 (0.75-0.94) | 0.90 (0.79-1.00) |
| MELD score | 0.70 (0.57-0.84) | 0.66 (0.53-0.79) | 0.68 (0.54-0.82) |
| CRP | 0.72 (0.58-0.85) | 0.69 (0.51-0.86) | 0.63 (0.40-0.86) |
| Relative lymphocyte count | 0.68 (0.53-0.82) | 0.61 (0.46-0.77) | 0.57 (0.40-0.74) |
| Neutrophil-to-lymphocyte ratio | 0.69 (0.53-0.83) | 0.60 (0.45-0.75) | 0.60 (0.45-0.75) |

Depicted are time-dependent AUCs with 95% confidence interval (95% CI) of pre-treatment suPAR levels, ALBI score, MELD score, CRP and relative lymphocyte count for the prediction of hepatic decompensation after 6, 12 and 18 months in cirrhotic patients with HCC receiving atezolizumab / bevacizumab. ALBI score, albumin-bilirubin score; CRP, C-reactive protein; MELD, model for end-stage liver disease; suPAR, soluble urokinase plasminogen activator receptor.

## Figure S3. SuPAR levels correlate with short- and long-term mortality in cirrhotic patients with HCC.


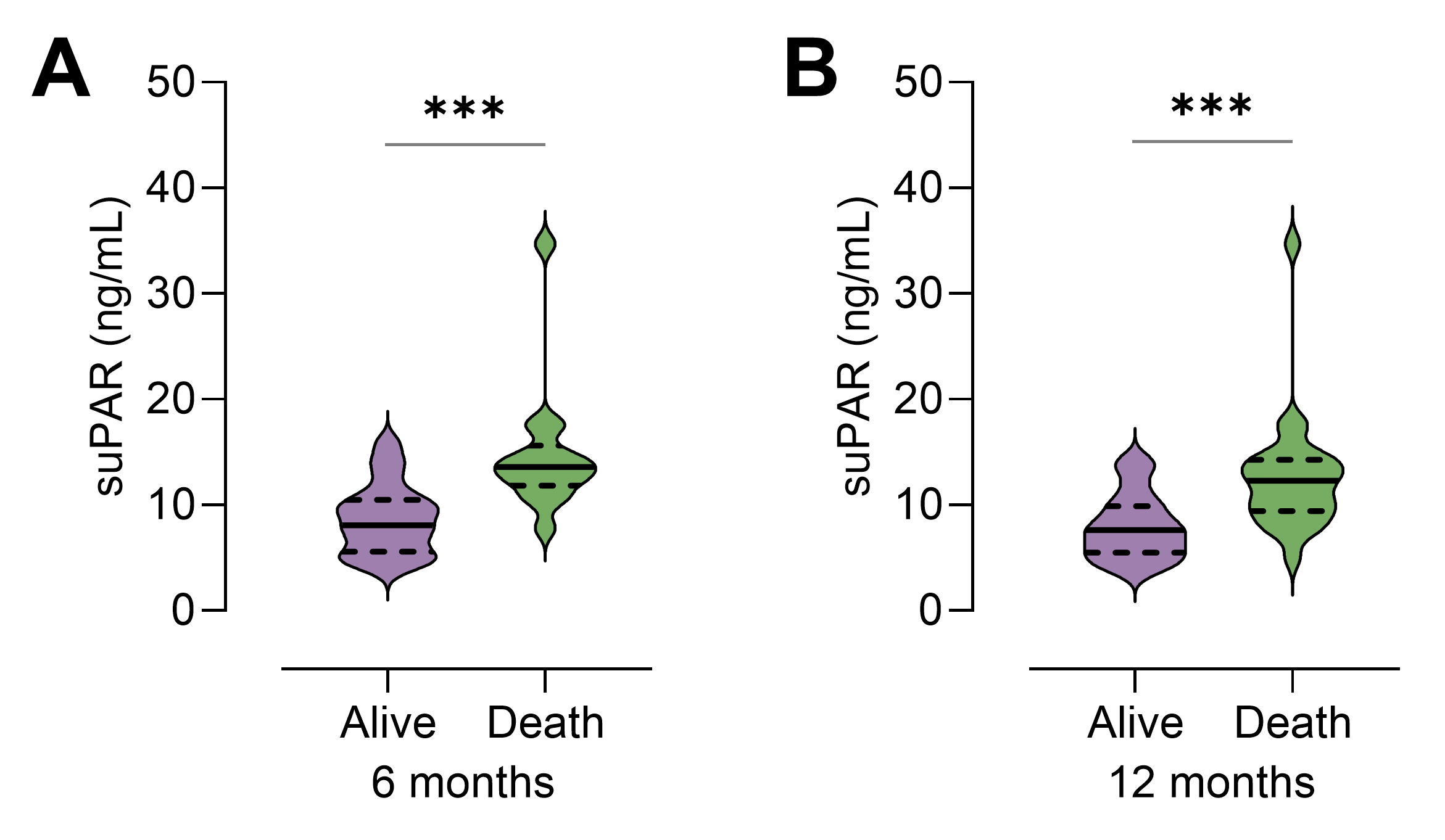

SuPAR levels were signiﬁcantly higher in patients who died within 6 months (A) and 12 months (B). Data are expressed as medians with IQR. p-values are from a non-parametric signed-rank test accounting for between and within group differences. *** p < 0.001. SuPAR, soluble urokinase plasminogen activator receptor.

## Table S5. Time-dependent AUCs for overall survival in cirrhotic patients with HCC.

| Biomarker | 6 months | 12 months | 18 months |
| --- | --- | --- | --- |
| suPAR | 0.86 (0.76-0.95) | 0.76 (0.63-0.88) | 0.76 (0.62-0.89) |
| ALBI | 0.71 (0.54-0.87) | 0.68 (0.56-0.81) | 0.69 (0.54-0.84) |
| MELD | 0.77 (0.64-0.91) | 0.61 (0.47-0.76) | 0.58 (0.45-0.72) |
| CRP | 0.81 (0.71-0.82) | 0.68 (0.53-0.83) | 0.56 (0.37-0.75) |
| Relative lymphocyte count | 0.82 (0.71-0.93) | 0.72 (0.57-0.87) | 0.72 (0.57-0.85) |
| Neutrophil-to-lymphocyte ratio | 0.79 (0.68-0.91) | 0.74 (0.62-0.88) | 0.74 (0.61-0.88) |

Depicted are time-dependent AUCs with 95% confidence interval (95% CI) of pre-treatment suPAR levels, ALBI score, MELD score, CRP and relative lymphocyte count for the prediction of mortality after 6, 12 and 18 months in cirrhotic patients with HCC receiving atezolizumab / bevacizumab. ALBI score, albumin-bilirubin score; CRP, C-reactive protein; MELD, model for end-stage liver disease; suPAR, soluble urokinase plasminogen activator receptor.

## Figure S4. Kaplan–Meier analysis of overall survival in cirrhotic patients with and without HCC.


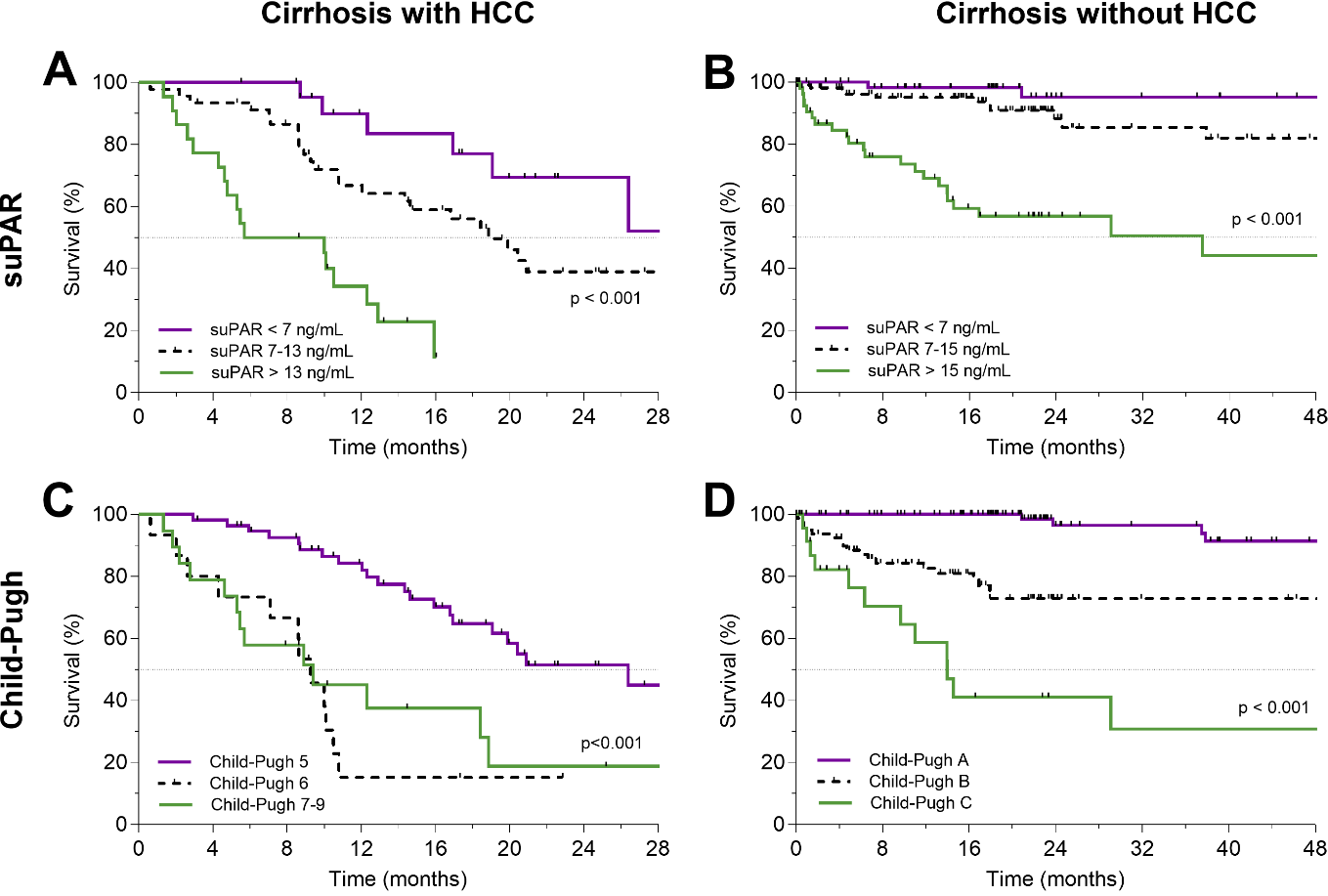

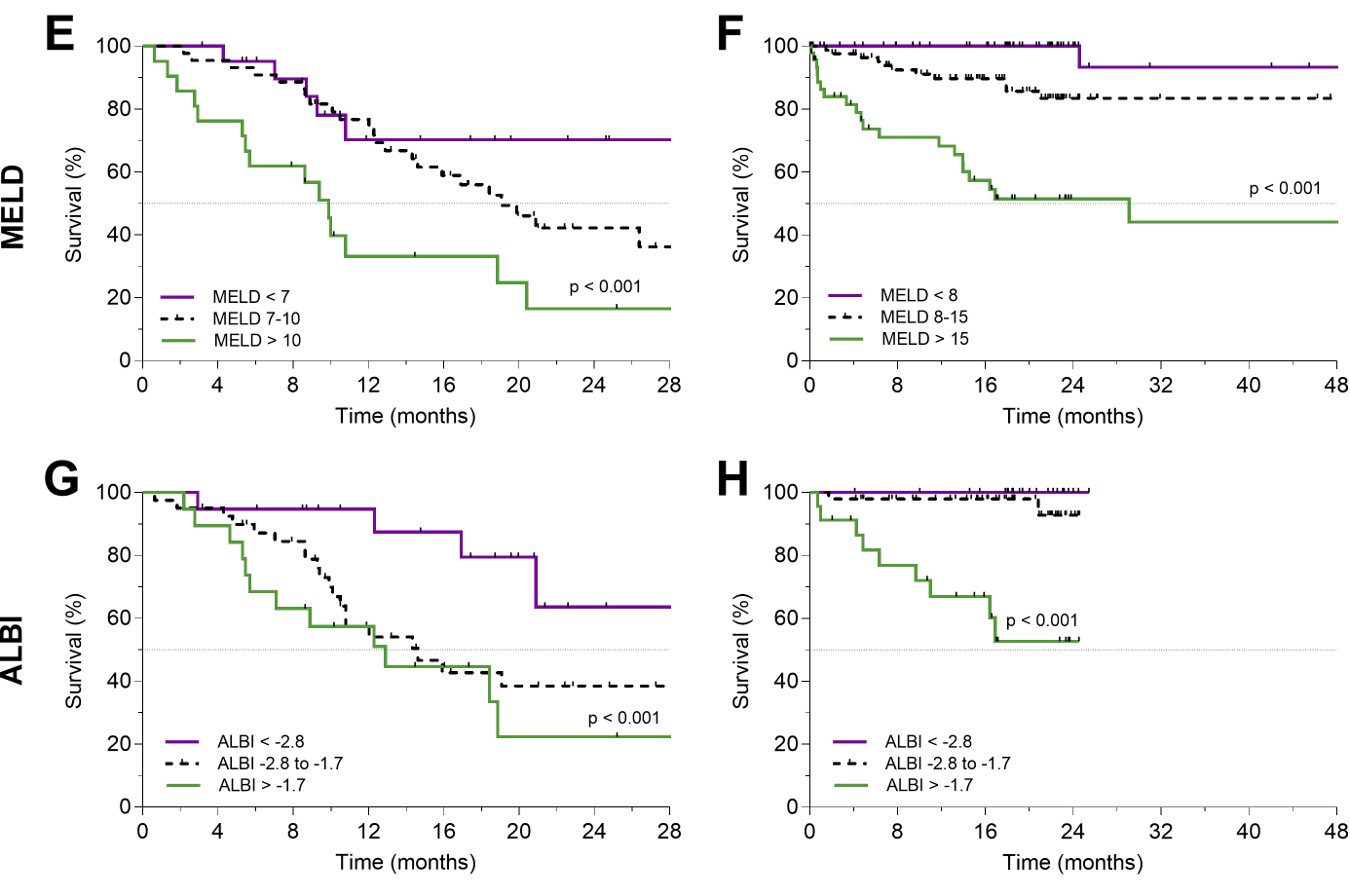


Depicted are Kaplan–Meier estimates of overall survival, risk-stratified by quartiles (Q1, Q2+3, Q4) of pre-treatment suPAR levels (A,B), Child-Pugh score (C,D), MELD score (E,F) and ALBI score (G,H) in cirrhotic patients with (A,C,E,G) and without HCC (B,D,F,H). Tick marks indicate censored data. p-values from log-rank test are given. ALBI score, albumin-bilirubin score; MELD, model for end-stage liver disease; suPAR, soluble urokinase plasminogen activator receptor.

## Table S6. Time-dependent AUCs for overall survival in cirrhotic patients without HCC.

| Biomarker | 6 months | 12 months | 18 months |
| --- | --- | --- | --- |
| suPAR | 0.82 (0.72-0.92) | 0.82 (0.73-0.91) | 0.84 (0.77-0.92) |
| ALBI | 0.87 (0.77-0.96) | 0.89 (0.82-0.97) | 0.96 (0.92-1.00) |
| MELD | 0.87 (0.81-0.94) | 0.86 (0.79-0.92) | 0.87 (0.81-0.93) |

Depicted are time-dependent AUCs with 95% confidence interval (95% CI) of pre-treatment suPAR levels, ALBI score, MELD score, CRP and relative lymphocyte count for the prediction of mortality after 6, 12 and 18 months in cirrhotic patients with HCC receiving atezolizumab / bevacizumab. ALBI score, albumin-bilirubin score; CRP, C-reactive protein; MELD, model for end-stage liver disease; suPAR, soluble urokinase plasminogen activator receptor.

##

## Table S7. Uni- and multivariate Cox-regression analyses for overall survival in cirrhotic patients without HCC.

|  | *Univariate regression models* | |  | *Multivariate regression models* | |
| --- | --- | --- | --- | --- | --- |
| **Parameter** | **p-value** | **Hazard ratio (95%CI)** |  | **p-value** | **Hazard ratio (95% CI)** |
| Male | 0.300 | 1.42 (0.73-2.74) |  |  |  |
| Age | 0.873 | 1.00 (0.97-1.02) |  |  |  |
| BMI | 0.127 | 1.07 (0.98-1.18) |  |  |  |
| Laboratory parameters |  |  |  |  |  |
| suPAR | <0.001 | 1.08 (1.06-1.11) |  | 0.287 | 1.05 (0.96-1.15) |
| Child-Pugh class | <0.001 | 4.09 (2.60-6.44) |  | 0.013 | 3.83 (1.33-11.04) |
| MELD score | <0.001 | 1.17 (1.12-1.23) |  | 0.783 | 0.98 (0.83-1.16) |
| Creatinine | 0.388 | 0.38 (0.04-3.41) |  |  |  |
| ALBI score | <0.001 | 9.00 (3.55-22.80) |  | 0.005 | 5.65 (1.68-18.96) |
| Etiology of liver cirrhosis |  |  |  |  |  |
| Viral | 0.769 | 1.15 (0.45-2.99) |  |  |  |
| Alcohol-related liver disease | 0.235 | 1.49 (0.77-2.88) |  |  |  |
| Metabolic dysfunction-associated steatohepatitis | 0.343 | 0.37 (0.05-2.89) |  |  |  |
| Relevant comorbidities |  |  |  |  |  |
| Chronic kidney disease | 0.856 | 1.16 (0.23-5.98) |  |  |  |

ALBI score, albumin-bilirubin score; BCLC, Barcelona Clinic liver cancer stage; BMI, body mass index; CRP, C-reactive protein; ECOG, Eastern Cooperative Oncology Group; MELD, model for end-stage liver disease; suPAR, soluble urokinase plasminogen activator receptor.

## Figure S5. Mortality risk according to different suPAR cut-off values.


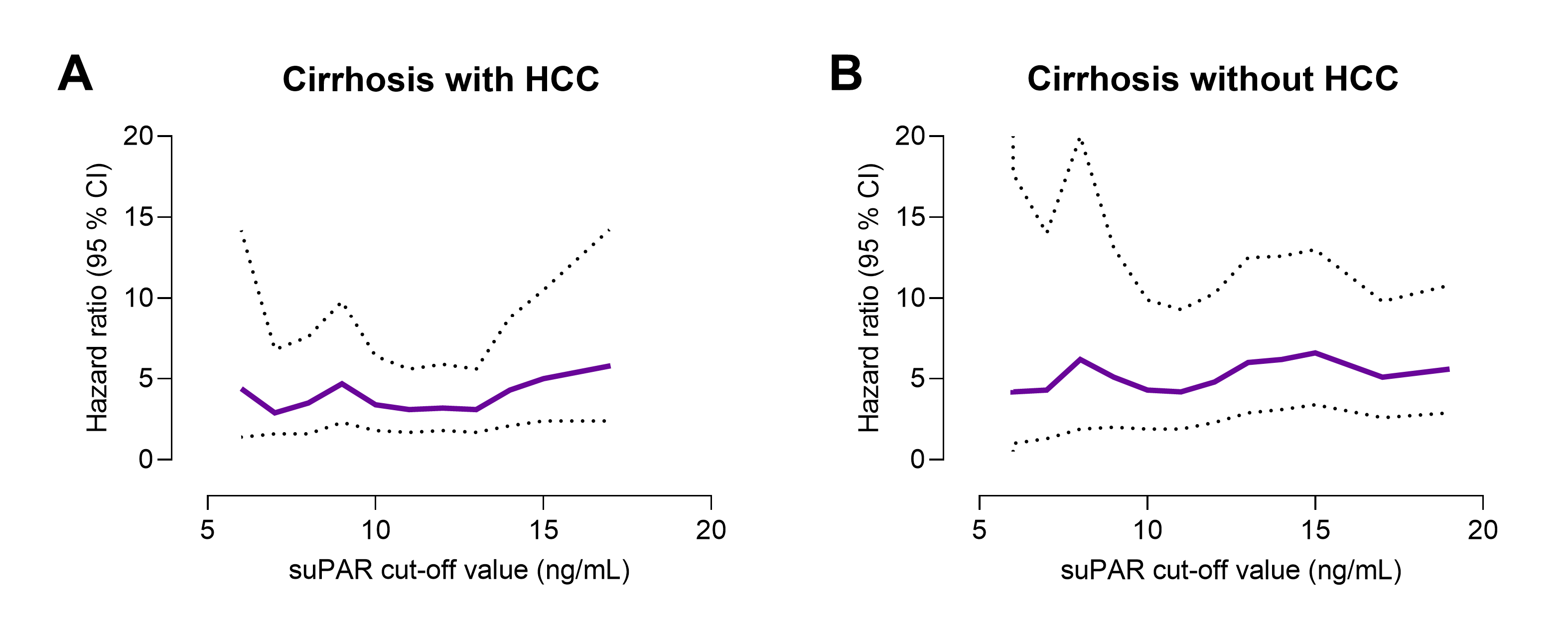
Depicted are hazard ratios with 95% confidence intervals (95% CI) from univariate Cox regression analysis for suPAR cut-off values across the entire measurement spectrum in cirrhotic patients with (A) and without HCC (B). SuPAR, soluble urokinase plasminogen activator receptor.

## Figure S6. Kaplan–Meier analysis of overall survival in patients with gastroenteropancreatic neuroendocrine neoplasia.


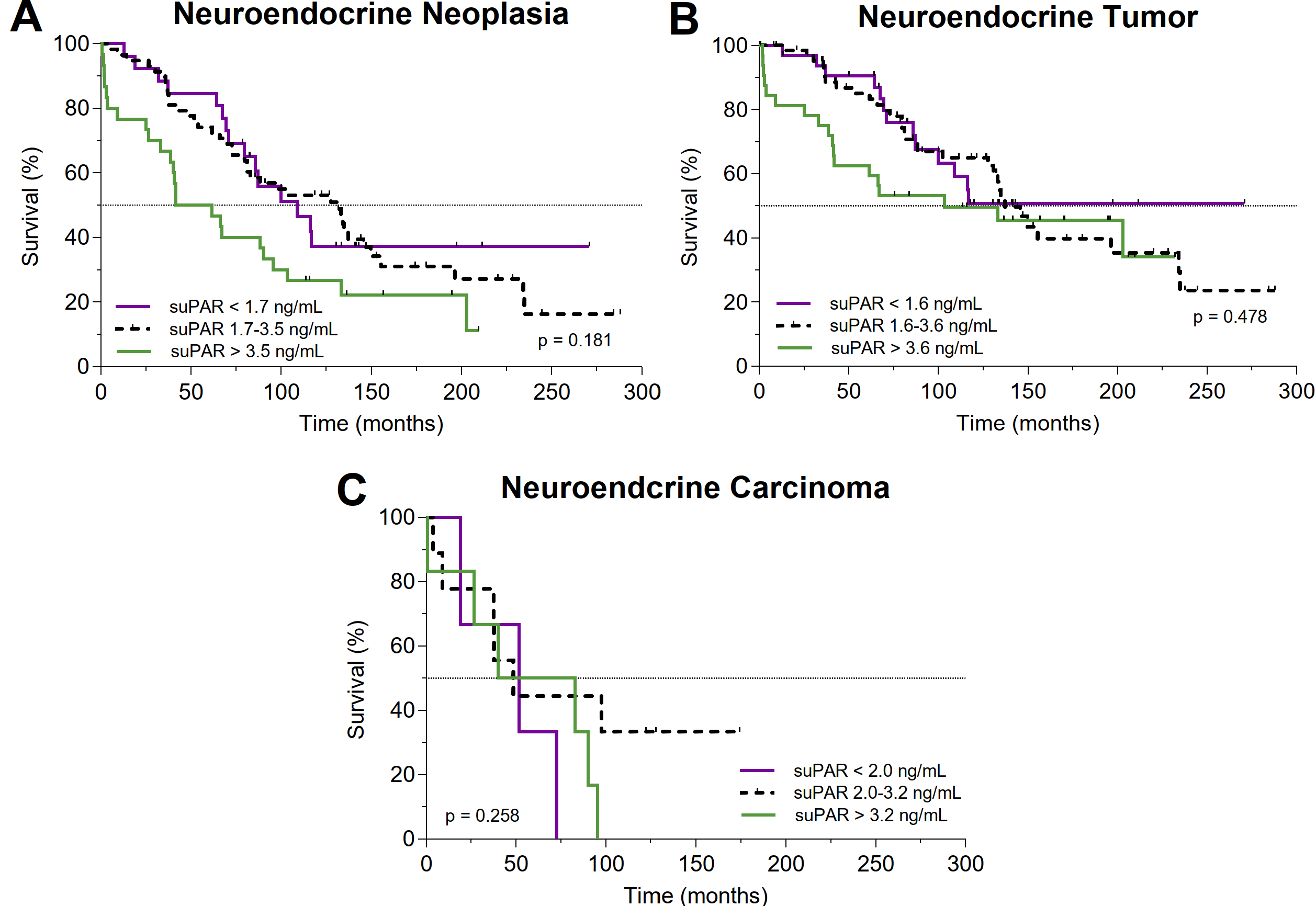
Depicted are Kaplan–Meier estimates of overall survival, risk-stratified by quartiles of pre-treatment suPAR levels (Q1, Q2+3, Q4) in patients with GEP-NEN (A), GEP-NET (B) and GEP-NEC (C). Tick marks indicate censored data. p-values from log-rank test are given. SuPAR, soluble urokinase plasminogen activator receptor.

## Figure S7. Kaplan–Meier analysis of overall survival according to HCC progression and hepatic decompensation in cirrhotic patients with HCC.

**
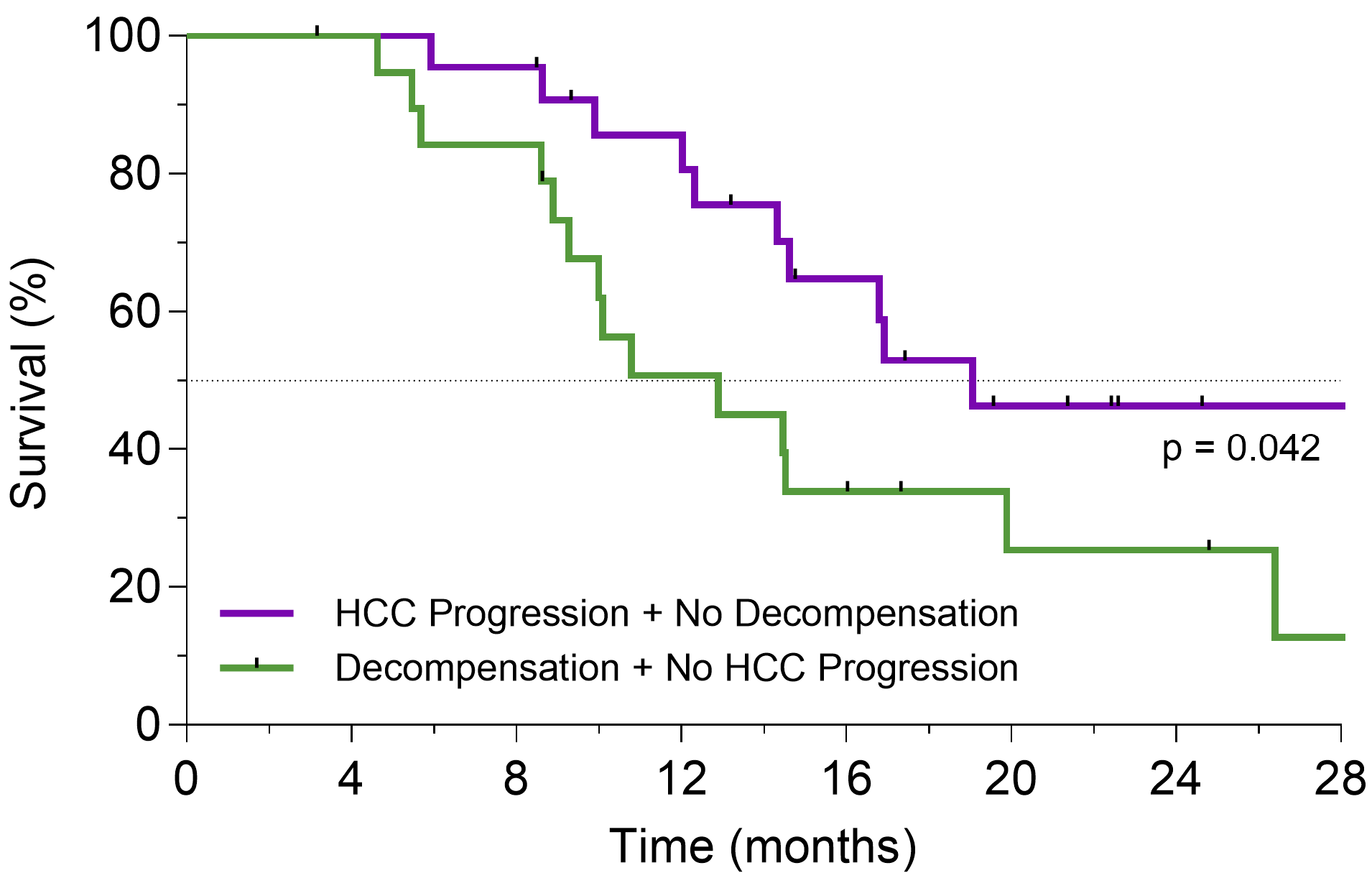
**Depicted are Kaplan–Meier estimates of overall survival in patients with HCC progression and without hepatic decompensation vs. patients without HCC progression and with hepatic decompensation. Tick marks indicate censored data. p-values from log-rank test are given.

## Figure S8. Kaplan–Meier analysis of overall survival according to treatment response, hepatic decompensation and suPAR levels in cirrhotic patients with HCC.


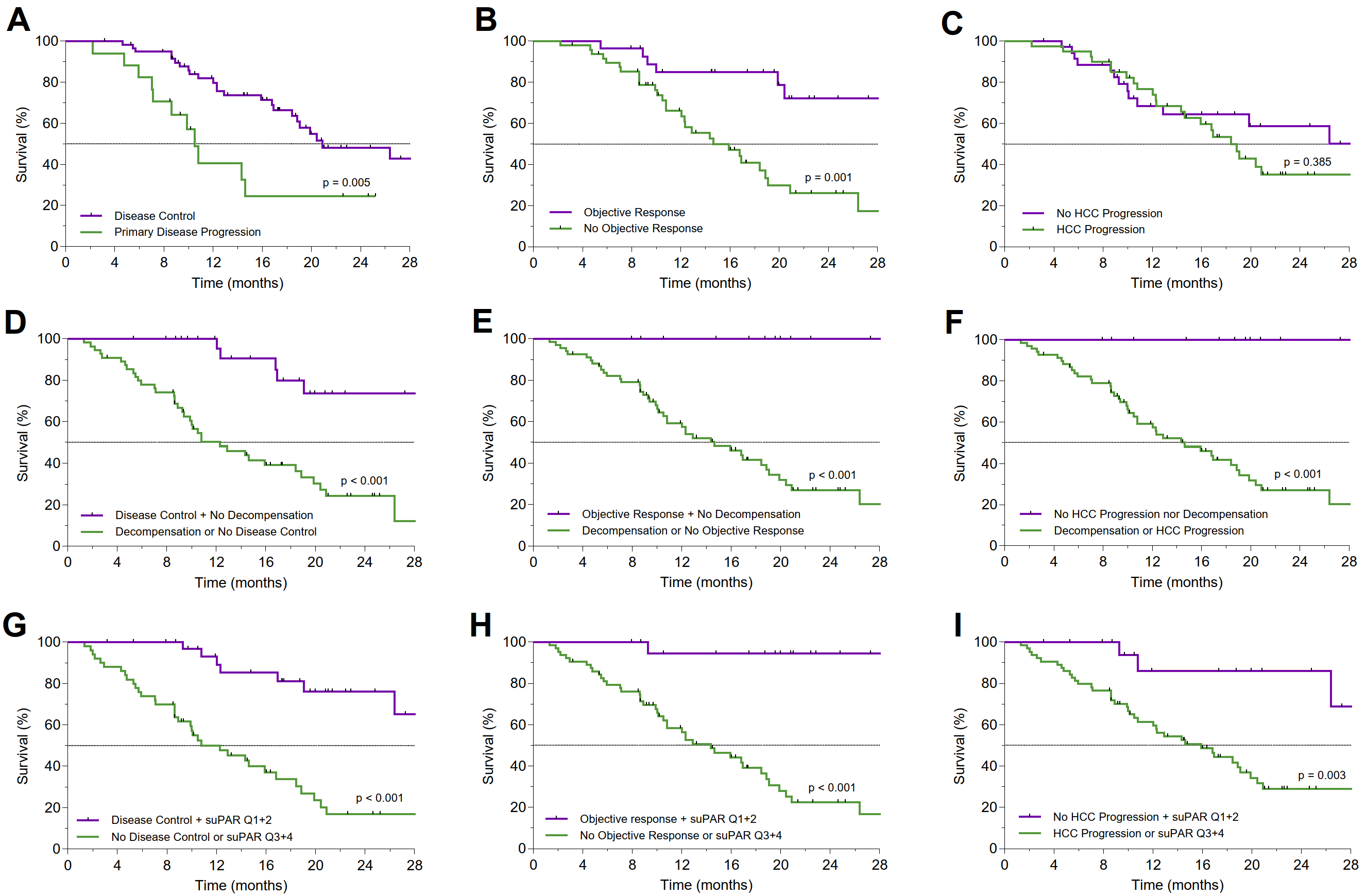


Depicted are Kaplan–Meier estimates of overall survival according to treatment response (A-C), treatment response plus hepatic decompensation (D-F) and treatment response plus suPAR quartiles (G-I). Tick marks indicate censored data. p-values from log-rank test are given. SuPAR, soluble urokinase plasminogen activator receptor.

## Figure S9. Kaplan–Meier analysis of overall survival and time without hepatic decompensation according to changes in suPAR levels in cirrhotic patients with HCC.


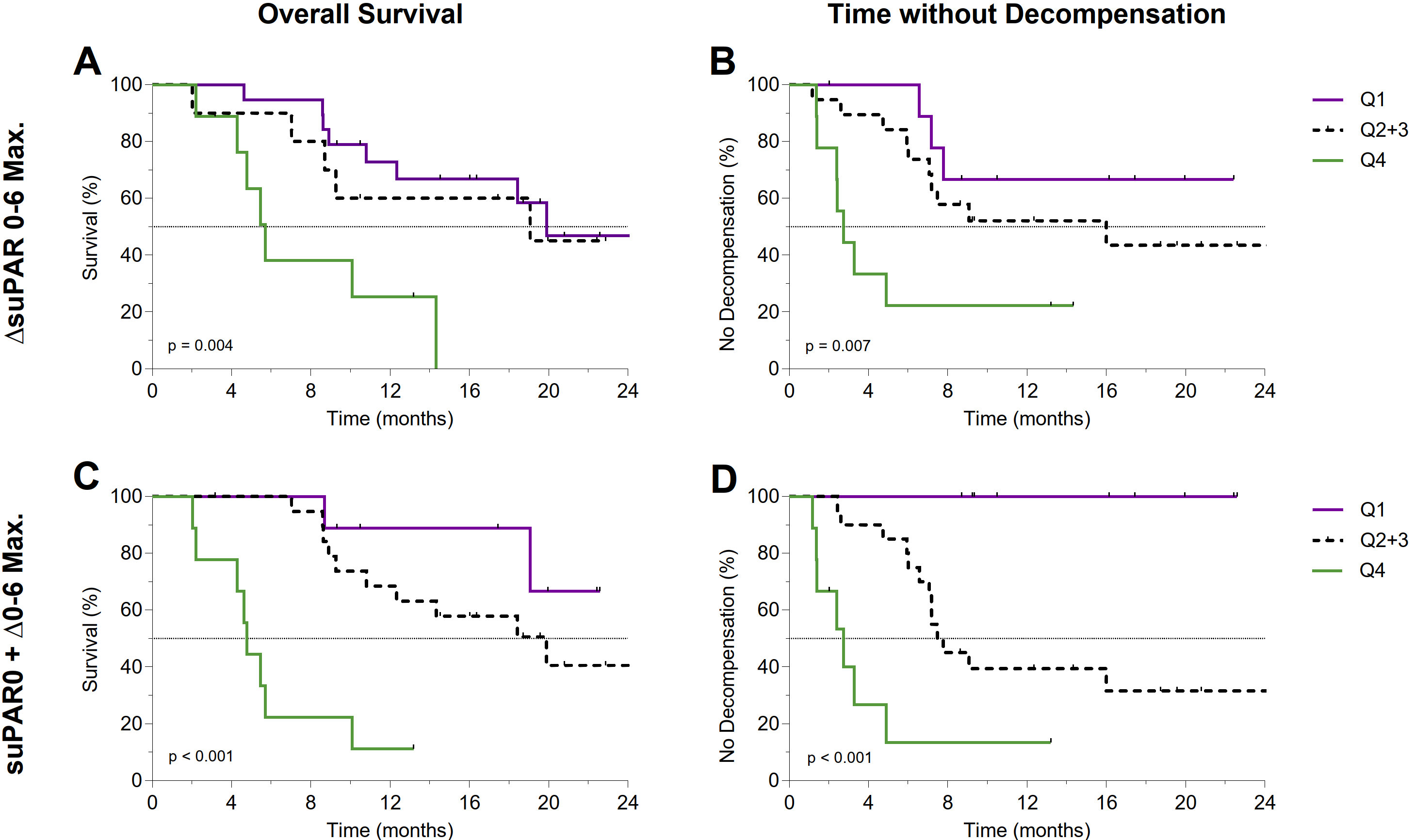


Depicted are Kaplan–Meier estimates of overall survival (A,C) and time without hepatic decompensation (B,D), risk-stratified by quartiles (Q1, Q2+3, Q4) of maximum change in suPAR levels during the first 6 months [∆suPAR 0-6 Max.] (A,B) and the combination of suPAR 0-6 Max. with pre-treatment suPAR levels (C,D). Logistic regression was used to combine pre-treatment suPAR concentrations with changes in suPAR levels. Tick marks indicate censored data. p-values from log-rank test are given. SuPAR, soluble urokinase plasminogen activator receptor.

## Figure S10. Kaplan–Meier analysis of overall survival and time without hepatic decompensation in patients with HCC and Child-Pugh A cirrhosis.


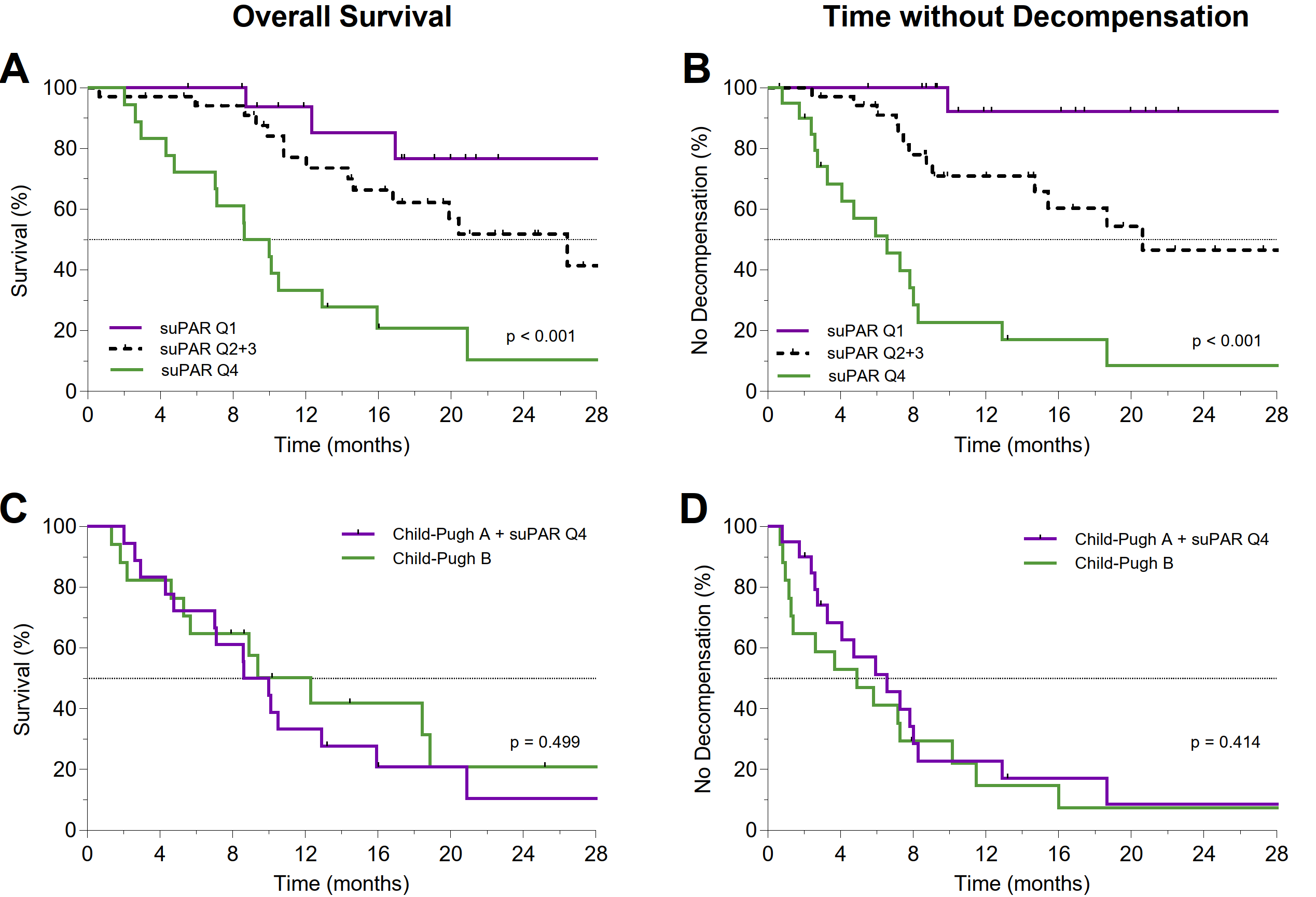
Depicted are Kaplan–Meier estimates of overall survival and time without hepatic decompensation, risk-stratified by quartiles of pre-treatment suPAR levels [Q1, Q2+3, Q4] (A,B) and comparing patients with Child-Pugh A cirrhosis and baseline suPAR levels in den fourth quartile versus Child-Pugh B patients (C,D). Tick marks indicate censored data. p-values from log-rank test are given. SuPAR, soluble urokinase plasminogen activator receptor.

##

## Figure S11. Child-Pugh class subgroup analysis according to ALBI grades in cirrhotic patients with HCC.


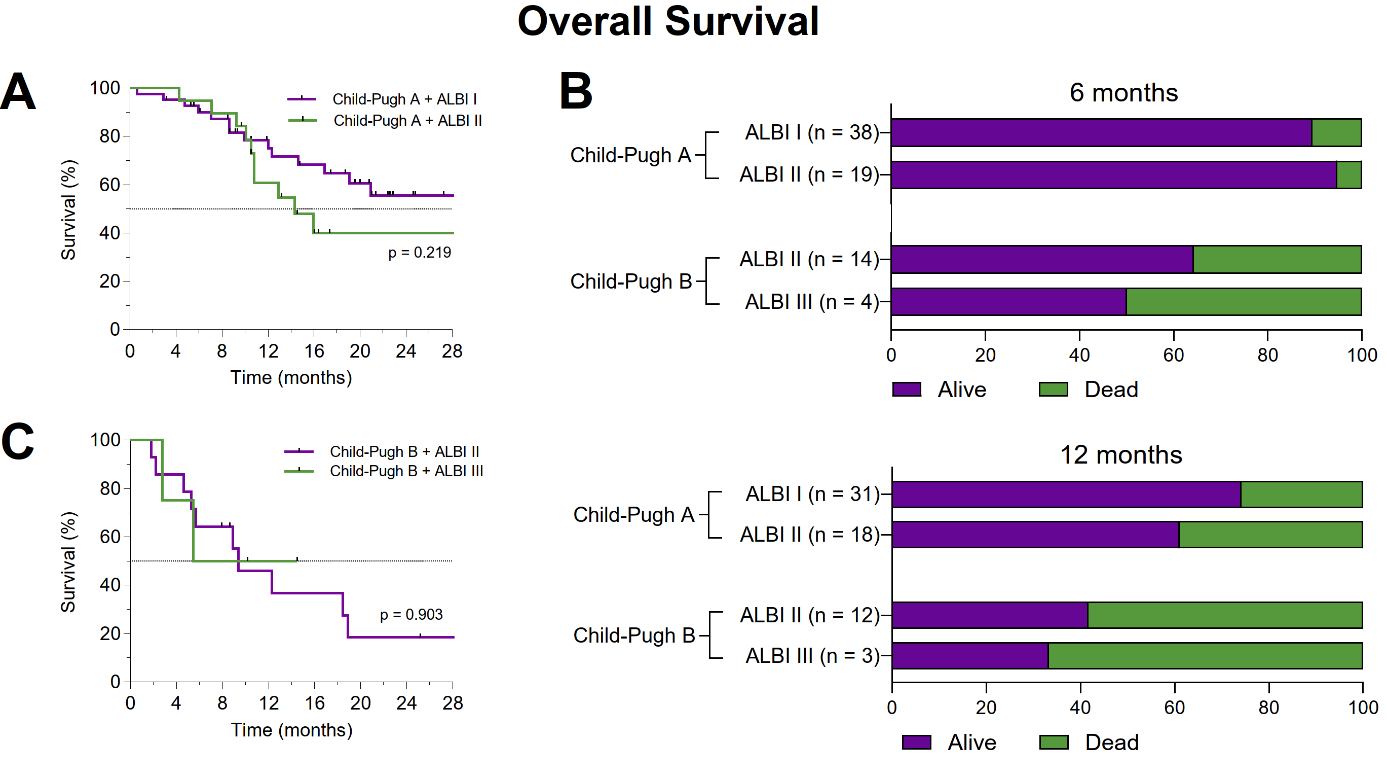

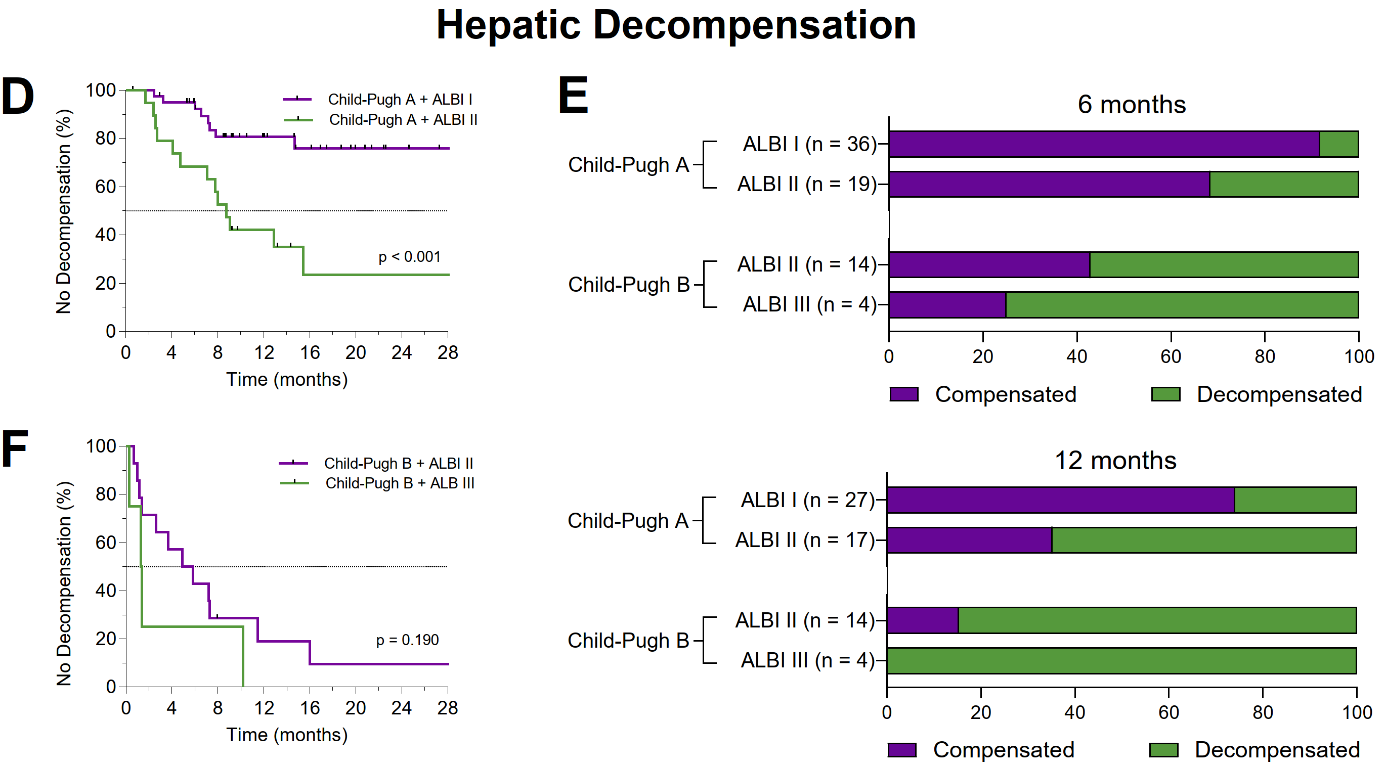


Depicted are Kaplan–Meier estimates of overall survival (A,C) and time without hepatic decompensation (D,F) according to Child-Pugh class and ALBI grades. (B,E) Stacked bar charts of percentages of deaths (B) and hepatic decompensations (E) after 6 and 12 months, stratified by Child-Pugh class and ALBI grades.
